# Sexual identity of enterocytes regulates autophagy to determine intestinal health, lifespan and responses to rapamycin

**DOI:** 10.1038/s43587-022-00308-7

**Published:** 2022-12-01

**Authors:** Jennifer C. Regan, Yu-Xuan Lu, Enric Ureña, Ralf L. Meilenbrock, James H. Catterson, Disna Kißler, Jenny Fröhlich, Emilie Funk, Linda Partridge

**Affiliations:** 1 Institute of Healthy Ageing, Department of Genetics, Evolution and Environment, University College London, London, UK; 2 Institute of Immunology and Infection Research, University of Edinburgh, Edinburgh, UK; 3 Max Planck Institute for Biology of Ageing, Cologne, Germany

**Keywords:** Metabolism, Drug delivery, Ageing

## Abstract

Pharmacological attenuation of mTOR presents a promising route for delay of age-related disease. Here we show that treatment of *Drosophila* with the mTOR inhibitor rapamycin extends lifespan in females, but not in males. Female-specific, age-related gut pathology is markedly slowed by rapamycin treatment, mediated by increased autophagy. Treatment increases enterocyte autophagy in females, via the H3/H4 histone-Bchs axis, whereas males show high basal levels of enterocyte autophagy that are not increased by rapamycin feeding. Enterocyte sexual identity, determined by *transformer*^*Female*^ expression, dictates sexually dimorphic cell size, H3/H4-*Bchs* expression, basal rates of autophagy, fecundity, intestinal homeostasis and lifespan extension in response to rapamycin. Dimorphism in autophagy is conserved in mice, where intestine, brown adipose tissue and muscle exhibit sex differences in autophagy and response to rapamycin. This study highlights tissue sex as a determining factor in the regulation of metabolic processes by mTOR and the efficacy of mTOR-targeted, anti-aging drug treatments.

## Abstract (web summary)

Understanding sex differential responses to geroprotective interventions is key to developing personalized longevity treatments. Here, Regan, Lu et al. show that the sexual identity of intestinal enterocytes regulates autophagy to determine the response to the drug rapamycin.

## Main

Sex differences in lifespan are almost as prevalent as sex itself^1,2^. Women are the longer-lived sex in humans, in some countries by an average of >10 years, and yet bear a greater burden of age-related morbidities than do men^3,4^. Many aspects of human physiology that affect homeostasis over the life course show profound sex differences, including metabolism^5^, responses to stress^6^, immune responses and autoinflammation^7–9^ and the rate of decline of circulating sex steroid hormones (menopause and andropause)^10^. These physiological differences lead to different risks of developing age-related diseases, including heart disease, cancer and neurodegeneration^11,12^. Sex differences can also determine responses to pharmacological treatments;^13^ potentially both acutely, by regulating physiology and metabolism, and chronically, by influencing the type and progression of tissue pathology. Understanding how sex influences the development of age-related disease and their responses to treatment will be key to moving forward with the development of geroprotective therapeutics.

Greater longevity in females than in males is prevalent across taxa^1,2,14^. Evolutionary drivers for sex differences in longevity include mating systems, physical and behavioral dimorphisms and consequent differences in extrinsic mortality, sex determination by heterogametism and mitochondrial selection^1,2,14,15^. Studies in laboratory model systems can help uncover the mechanisms leading to sexual dimorphism in longevity. Lifespan is a malleable trait, and genetic, environmental and pharmacological interventions can ameliorate the effects of aging. These interventions often target highly conserved, nutrient-sensing signaling pathways, and their effects are frequently sex specific^13,16^. Dietary restriction extends lifespan more in female than in male *Drosophila melanogaster*, at least in part by targeting a dimorphic decline in gut physiology, which is much more evident in females^17^. Dietary restriction influences nutrient sensing pathways such as insulin/Igf (IIS)/mTOR, and targeting these pathways directly offers a more translational route for anti-aging therapy than do chronic dietary regimens^18–21^.

mTOR is a highly conserved signaling hub that integrates multiple cues to regulate key cellular functions, including cell growth, division, apoptosis and autophagy. The mTOR complex 1 (mTORC1) is activated by both nutrients and growth factors such as epidermal growth factor and IIS ligands, via phosphoinositide 3-kinase and Akt, such that it responds to both organismal and intracellular energy status^22^. Attenuation of mTORC1 activity genetically by a null mutation in the mTORC1 substrate ribosomal protein *S6 kinase beta-1* (*S6K1*) gene increases lifespan in female, but not male, mice^23^. Pharmacological inhibition of mTORC1 by rapamycin is currently the only pharmacological intervention that extends lifespan in all major model organisms^18,20,24^. Treatment of genetically heterogenous mice induced lifespan extension, to a greater extent in females than in males^25,26^. Interestingly, a subsequent study demonstrated sexually dimorphic effects on cancer incidence and type^27^. The physiological bases for these dimorphic responses are not well understood.

Chronic treatment with rapamycin extends lifespan substantially more in female *Drosophila melanogaster* than in males^28^ and attenuates development of age-related gut pathologies in *Drosophila* females^29^. However, the effect of rapamycin on aging pathology in *Drosophila* males is unknown. Here, we show that treatment with rapamycin extends lifespan in female flies only. Intestinal ageing in females is attenuated by rapamycin treatment, through upregulation of autophagy in enterocytes. There are strong dimorphisms in baseline metabolic regulation of intestinal cells, whereby male enterocytes appear to represent an intrinsic, minimal limit for cell size and an upper limit for autophagy, neither of which are pushed further by rapamycin treatment. By manipulating genetic determination of tissue sex, we show that sexual identity of enterocytes determines physiological responses to mTOR attenuation, including homeostatic maintenance of gut health and function, and lifespan, through autophagy activation by the histones–Bchs axis^30^. Furthermore, we demonstrate sexual dimorphism in basal autophagy and in response to rapamycin in mouse tissues, including the jejunum and colon of the intestine. These data show the importance of cellular sexual identity in determining baseline metabolism, consequent rates of tissue aging and responses to anti-aging interventions.

## Results

### Rapamycin treatment extends lifespan in females, but not in males

**Fig. 1:**
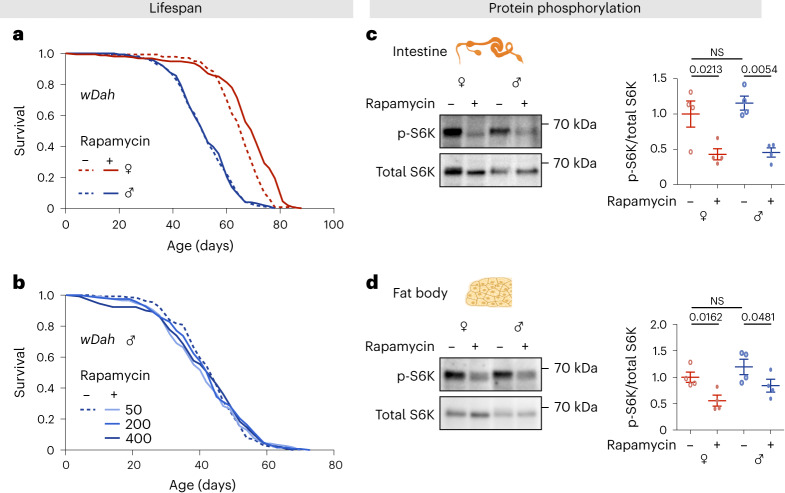
Rapamycin treatment extends lifespan in *w*^*Dah*^ females only but reduces phosphorylation of S6K in both sexes. **a**, Adult-onset rapamycin treatment (200 µM) extended the lifespan of *w*^*Dah*^ females, but not males (log-rank test, females *P* = 2.1E-06, males *P* = 0.77, *n* = 143–171 flies per condition). See also Supplementary Table 1. **b**, Adult-onset rapamycin treatment at three concentration (50, 200 and 400 µM) did not extend the lifespan of *w*^*Dah*^ males (log-rank test, 50 µM *P* = 0.60, 200 µM *P* = 0.75, 400 µM *P* = 1, *n* = 118–131 flies per condition). See also Supplementary Table 2. **c**,**d**, The level of p-S6K in the intestine and the fat body was substantially reduced by rapamycin treatment (200 µM) in both females and males at 10 days of age (*n* = 4 biological replicates of 10 intestines per replicate, two-way analysis of variance (ANOVA), interaction *P* > 0.05; post-hoc test). Data are presented as mean values ± standard error of the mean (s.e.m.). NS, not significant. Source data

**Extended Data Fig. 1:**
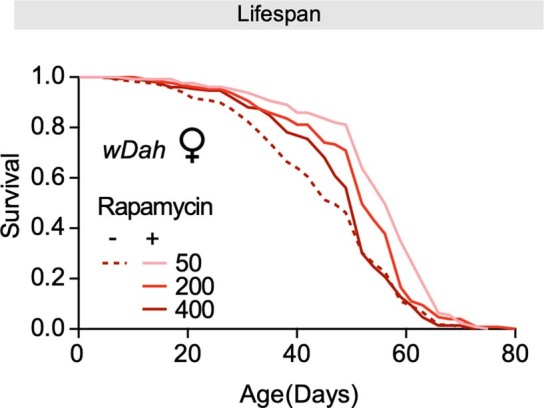
Rapamycin treatment extends lifespan in w^Dah^ females. Adult-onset rapamycin treatment in all three tested concentration (50, 200 and 400 µM) extended the median lifespan of *w*^*Dah*^ females. (log-rank test, 50 µM p = 9.0E-08, 200 µM p = 1.2E-03, 400 µM p = 0.04, n = 118-150 flies per condition). See also Supplementary Table 2. Source data

**Extended Data Fig. 2:**
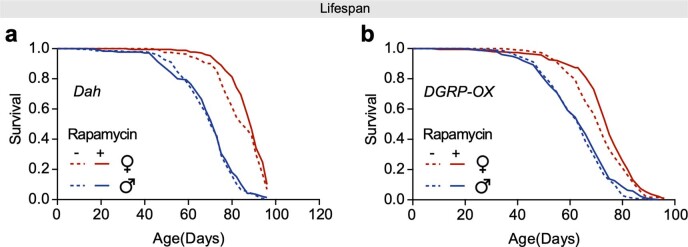
Rapamycin treatment extends lifespan in *Dah* and *DGRP* females only. **a**, Adult-onset rapamycin treatment (200 µM) extended the lifespan of *Dah* females but not males (log-rank test, females p = 0.039, males p = 0.73, n = 188-201 flies per condition). **b**, Adult-onset rapamycin treatment (200 µM) extended the lifespan of females but not males of an outbred, genetically heterogenous fly line (*DGRP-OX*) (log-rank test, females p = 0.010, males p = 0.23, n = 181-189 flies per condition). See also Supplementary Table 3. Source data

**Extended Data Fig. 3:**
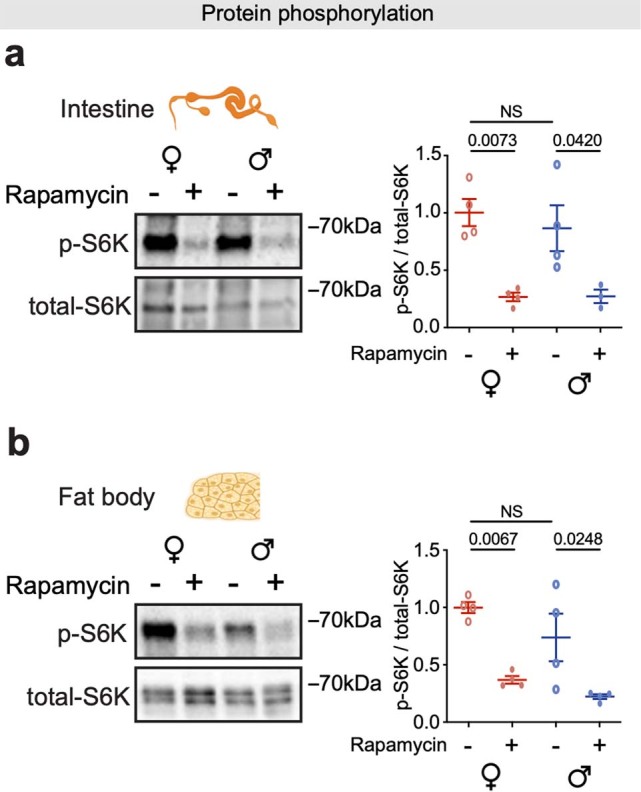
Rapamycin treatment reduces phosphorylation of S6K in both sexes. **a,b**, The level of phospho-S6K in the intestine and the fat body was substantially reduced by rapamycin treatment (200 µM) both in females and males, at 45 days of age (n = 4 biological replicates of 10 intestines per replicate, two-way ANOVA, interaction p > 0.05; post-hoc test). Data are presented as mean values  ± s.e.m. Source data

We treated adult *w*^*Dah*^ flies of both sexes with 200 μM rapamycin added to the food medium. At this dose, females, as expected^28^, showed a significant increase in lifespan, whereas males did not (Fig. 1a). Given that male flies eat less than females^31,32^ and hence may ingest less of the drug, we fed females and males rapamycin at three concentrations: 50, 200 and 400 μM. Females showed significantly extended lifespan at all three doses of the drug (Extended Data Fig. 1), but males showed no increase at any dose (Fig. 1b). To test if this finding generalized across fly genotypes, we also tested the *Dahomey (Dah)* line (from which *w*^*Dah*^ was originally derived), and a genetically heterogenous fly line derived from all lines that make up the *Drosophila Genetic Resource Panel* (*DGRP-OX*)^33^ and again observed significant lifespan extension only in females (Extended Data Fig. 2a,b). Inhibition of mTOR by rapamycin may, therefore, confer a beneficial effect in females that is absent in males. Alternatively, any beneficial physiological effects in males may be counteracted by negative effects, or males may be unable to respond to rapamycin. To determine if male tissues are sensitive to inhibition of mTORC1 by rapamycin, we measured phosphorylated S6K (p-S6K) levels in dissected intestines and fat body tissue at 10 days (Fig. 1c,d) and 45 days of age (Extended Data Fig. 3a,b). Both sexes showed a significant reduction in p-S6K levels in intestine and fat body in response to rapamycin, with no significant interaction between sex and treatment. The dimorphic response of lifespan to rapamycin was therefore probably not due to sex differences in suppression of mTORC1 signaling by the drug.

### Age-related gut pathology is reduced in females treated with rapamycin

**Fig. 2:**
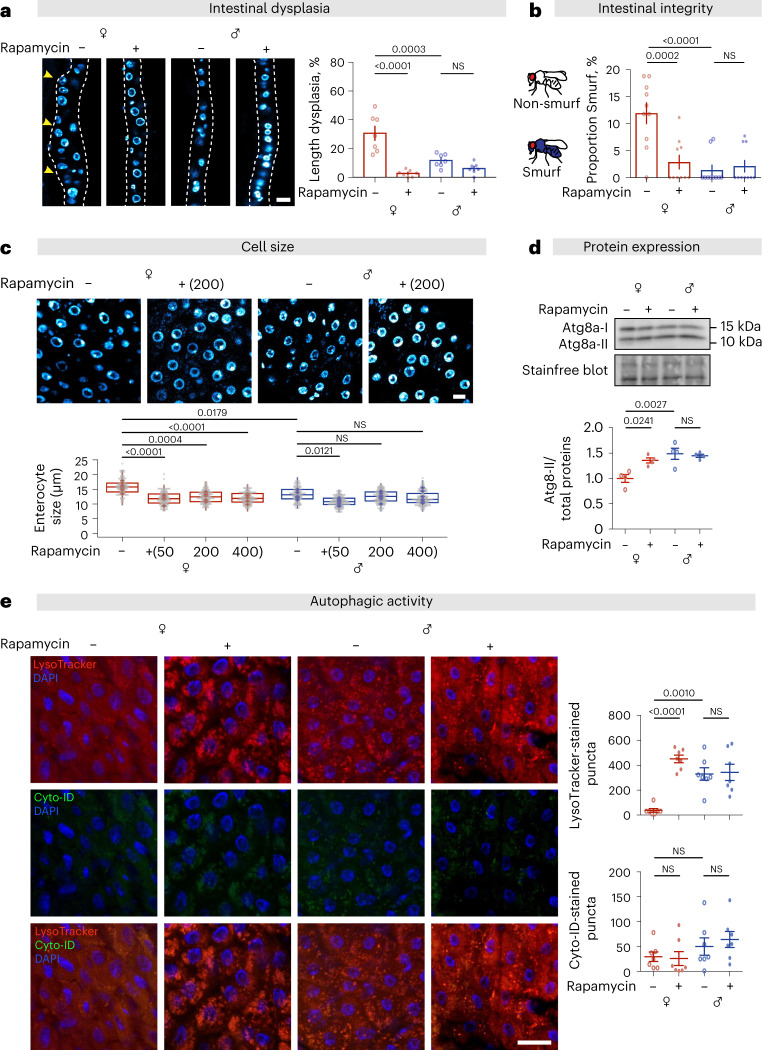
Rapamycin treatment reduces age-related gut pathology and enterocyte size and elevates autophagy and barrier integrity in *w*^*Dah*^ females, but not in males. **a**, Females showed greater age-related dysplasia in aged guts, which was attenuated by rapamycin treatment (200 µM), at 50 days of age (scale bar = 15 µm; *n* = 7 intestines, two-way ANOVA, interaction ***P < 0.001; post-hoc test). **b**, A higher number of female flies suffered barrier function decline (Smurf phenotype) than did males, and showed increased barrier function in response to rapamycin (200 µM), at 60 days of age (bar charts show *n* = 10 biological replicates of 10–19 flies per replicate, two-way ANOVA, interaction *P* < 0.001; post-hoc test). **c**, Cell size of enterocytes in females was larger than in males, and reduced to the same size as in males in response to rapamycin treatment (50, 200 and 400 µM), at 10 days of age (scale bar = 10 µm; *n* = 6–8 intestines, *n* = 10–20 enterocytes per intestine; circles indicate individual values, and diamonds represent the average value per intestine; linear mixed model, interaction *P* < 0.01; post-hoc test). **d**, The expression of Atg8a-II in the gut of females was lower than in males, and rapamycin treatment (200 µM) increased it to a similar level as in males, at 10 days of age (*n* = 4 biological replicates of 10 intestines per replicate, two-way ANOVA, interaction *P* < 0.01; post-hoc test). **e**, The number of LysoTracker-stained puncta in the gut of females was lower than in males, and rapamycin (200 µM) increased it to the level measured in males. Neither sex nor rapamycin had an effect on the number of Cyto-ID-stained puncta in the intestine, at 10 days of age (scale bar = 20 µm; *n* = 7 intestines per condition; *n* = 2-3 pictures per intestine; data points represent the average value per intestine; linear mixed model, interaction LysoTracker-stained puncta, *P* < 0.001, Cyto-ID-stained puncta, *P* > 0.05; post-hoc test). Data are presented as mean values ± s.e.m. For box-and-whiskers plot (c), median, 25th and 75th percentiles, and Tukey whiskers are indicated. Source data

**Extended Data Fig. 4:**
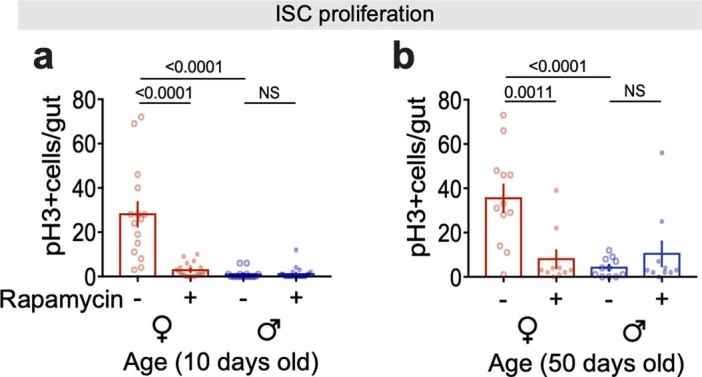
Rapamycin treatment reduces ISC mitoses in *w*^*Dah*^ females but not in males. **a,b**, Rapamycin treatment (200 µM) reduced the number of pH3 + cells in females, to a similar level as in untreated males, while it did not affect the number of pH3 + cells in males, (**a**) at 10 days of age, and (**b**) at 50 days of age (10 days n = 15-24 intestines, 50 days n = 10-12 intestines, two-way ANOVA, interaction 10 days p < 0.001, 50 days p < 0.01; post-hoc test). Data are presented as mean values ± s.e.m. Source data

Dietary restriction attenuates female-specific, age-related intestinal pathologies in *Drosophila*, leading to a greater extension of lifespan in females than in males^17^. We therefore investigated the effect of rapamycin on age-related decline in the structure and function of the gut. Dysplastic pathology can be quantified by assessing the proportion of the intestinal epithelium that is no longer maintained as a single layer^30,34^. In parallel, gut barrier function can be assessed using well-described methods to detect the onset of gut leakiness^35,36^. As previously reported^17,29,30^, females treated with rapamycin showed a strong attenuation of epithelial pathology (Fig. 2a) and intestinal stem cell (ISC) mitoses^37^ (Extended Data Fig. 4a,b), in parallel with better maintenance of barrier function assessed by extra-intestinal accumulation of blue dye added to food (the ‘Smurf’ phenotype)^35,38^ (Fig. 2b). In contrast, male flies showed only low levels of ISC mitoses and intestinal pathology, and these effects were not reduced by rapamycin treatment (Fig. 2a,b and Extended Data Fig. 3a,b)^39^.

### The microbiome does not change upon rapamycin treatment

**Extended Data Fig. 5:**
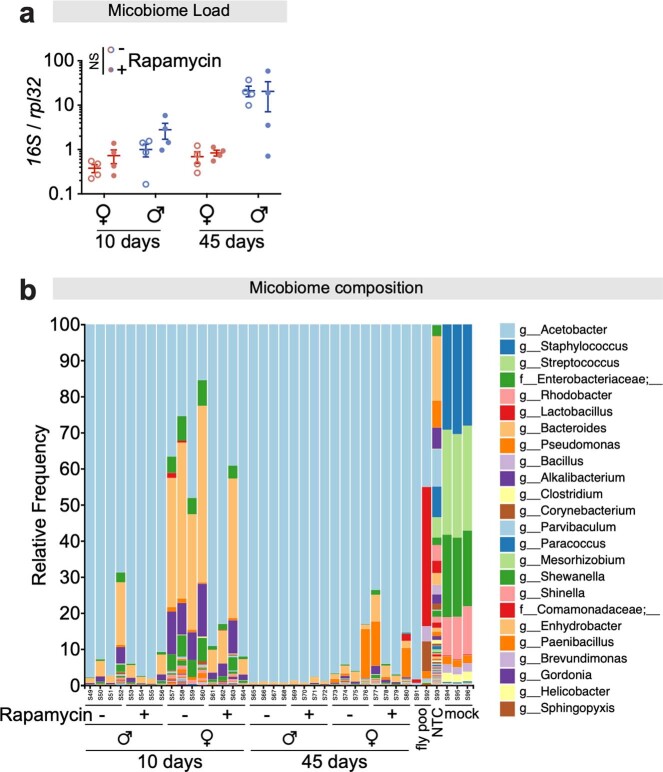
The microbiome does not change upon treatment with rapamycin. **a**, Bacterial load in intestines of *w*^*Dah*^ flies changed with age and sex, but was not affected by rapamycin treatment (200 µM) (n = 4 biological replicates of 10 intestines per replicate, three-way ANOVA, age p < 0.001, sexes p < 0.001, treatment p > 0.05). Data are presented as mean values ± s.e.m. **b**, Bacterial composition in intestines of *w*^*Dah*^ flies changed with age and sex, but was not affected by rapamycin treatment (200 µM). (n = 4 biological replicates of 10 intestines per replicate, PERMANOVA (the number of permutations = 999), treatment p > 0.05). Source data

Age-related shifts in the luminal microbial community can drive epithelial pathology in female *Drosophila*, through expansion of pathogenic bacterial species at the expense of commensals^38^. Attenuation of the mTOR pathway by rapamycin influences composition of the microbiome in mammals^27^. However, recent data demonstrated that chronic rapamycin treatment did not affect the microbiome in *Drosophila* females, at least under certain laboratory and diet conditions^40^. To investigate a role for the bacterial microbiome in mediating sex differences in the responses to rapamycin under our culture conditions, we sequenced the gut microbiome in young- and middle-aged flies of both sexes treated chronically with rapamycin. We found significant sex dimorphisms in load and composition of the microbiota (Extended Data Fig. 5a,b), which interacted with age. The load in old male flies increased by an order of magnitude compared with young male flies (Extended Data Fig. 5a). This increase was confirmed by quantifying *Acetobacter pomorum* transcripts relative to a *Drosophila* standard. No comparable increase was seen in females, either by assessing overall load, or load of *A. pomorum*. Rapamycin treatment did not significantly affect either load or composition in either sex (Extended Data Fig. 5a,b), suggesting that the sexually dimorphic effects of rapamycin treatment were not achieved through remodeling of the microbiome.

### Intestinal cell size is reduced in females, but not in males, upon rapamycin treatment

TOR plays a central role in regulating antagonistic anabolic and catabolic processes, and inhibition by rapamycin concomitantly decreases cell size and upregulates autophagy^41,42^. We fed rapamycin at doses between 50 μM and 400 μM and measured cell size after 14 days (Fig. 2c). Enterocyte size in untreated males was significantly smaller than in untreated females, as expected^17^, and was not significantly responsive to rapamycin treatment (Fig. 2c). In contrast, treatment at 50 µM reduced enterocyte size in females, to a size approximately 75% of that of control females and very similar to that of untreated males (Fig. 2c), with no further reduction at 4× (200 µM) or 8× (400 µM) higher doses.

### Male enterocytes have higher levels of basal autophagy that are not further increased by rapamycin treatment

Inhibition of mTORC1 by nutrient starvation, stress or pharmacological inhibition increases autophagy^22,41^. Autophagy can be measured in vivo in several ways, including western blot analysis of the lipidated form of the Atg8a protein (Atg8a-II), the fly ortholog of mammalian LC3. There was a sex dimorphism in basal levels of autophagy, with Atg8a-II protein levels higher in dissected intestines from untreated males than females (Fig. 2d). Rapamycin treatment substantially increased Atg8a-II in female intestines to levels similar to those in untreated males, whereas it had no significant effect on males (Fig. 2d). We performed co-stainings with LysoTracker and Cyto-ID, which selectively label autophagic vacuoles, to assess the autophagic flux. An increased number of LysoTracker puncta indicates that autophagic flux is increased or blocked, while an increase in the number of Cyto-ID puncta indicates that flux is blocked^30,43,44^. The number of LysoTracker-stained puncta, labelling autophagic vacuoles, was lower in untreated female intestines than in males (Fig. 2e) and when treated with rapamycin increased to levels that did not differ significantly from the basal level in males, whereas there was no measurable increase in male intestines (Fig. 2e). Neither sex nor rapamycin treatment affected the number of Cyto-ID puncta (Fig. 2e), suggesting that autophagic flux was not blocked. Taken together, these results demonstrate that males had higher basal levels of autophagy than did females and that only in females was there an increase in response to rapamycin treatment, which increased autophagy to similar levels to those seen in males.

### Suppressing autophagy in enterocytes reduces barrier function and decreases lifespan in males

**Fig. 3:**
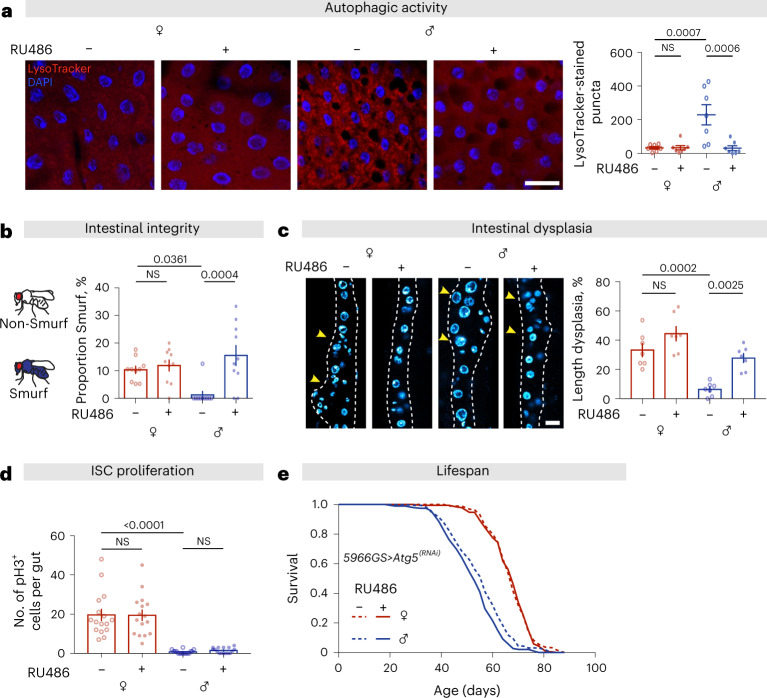
Autophagy in gut enterocytes regulates gut pathologies and lifespan. **a**, Adult-onset knockdown of *Atg5* in adult ECs (*5966GS* > *Atg5*^*[RNAi]*^) did not affect the number of LysoTracker-stained puncta in the gut of females, but decreased it in the gut of males to the level observed in females, at 20 days of age (scale bar = 20 µm; *n* = 7 intestines per condition; *n* = 2–3 pictures per intestine, data points represent the average value per intestine; linear mixed model, interaction *P* < 0.01; post-hoc test). **b**, Females had higher gut leakiness (number of Smurfs) than males, and adult-onset knockdown of *Atg5* in adult ECs in males significantly increased it, to the level observed in females, at 60 days of age (bar charts show *n* = 10 biological replicates of 8–20 flies per replicate, two-way ANOVA, interaction *P* < 0.01; post-hoc test). **c**, Adult-onset knockdown of *Atg5* in adult ECs did not affect the level of dysplasia in the gut of females, but increased it in the gut of males to the level observed in females, at 50 days of age (scale bar = 15 µm; *n* = 7 intestines, two-way ANOVA, interaction *P* > 0.05; post-hoc test). **d**, Adult-onset knockdown of *Atg5* in adult ECs did not change the number of pH3^+^ cells in either females or males, at 20 days of age (*n* = 16 intestines, two-way ANOVA, interaction *P* > 0.05; post-hoc test). **e**, Adult-onset knockdown of *Atg5* in adult ECs shortened lifespan of males, but not females (log-rank test, females *P* = 0.80, males *P* = 4.5 × 10^−3^, *n* = 199 flies per condition). See also Supplementary Table 4. Data are presented as mean values ± s.e.m. Source data

To probe the role of increased basal autophagy levels in males, we genetically suppressed the process, by expressing RNA interference (RNAi) against the essential autophagy gene *Atg5* in adult enterocytes (ECs), using the Geneswitch system^45^, *5966GS* > *Atg5*^*[RNAi]*^. In line with our previous result (Fig. 2e), males showed markedly higher basal levels of intestinal autophagy than did females (Fig. 3a). Knockdown of *Atg5* reduced autophagy in males to similar levels as in females, whereas females showed no response (Fig. 3a).

Autophagy maintains homeostasis of ageing tissues, and its manipulation can affect lifespan^46,47^. Indeed, gut barrier function was reduced in aged male flies with suppressed autophagy, to levels similar to those seen in females (Fig. 3b). In contrast, expression of *Atg5*^*[RNAi]*^ had no effect on barrier function in female flies (Fig. 3b), likely due to their already low levels of intestinal autophagy. Development of dysplasia was also significantly increased in aged *5966GS* > *Atg5*^*[RNAi]*^ males compared to controls, but not in females (Fig. 3c). When we analyzed ISC proliferation at 20 days, we did not see an upregulation of mitoses in male *5966GS* > *Atg5*^*[RNAi]*^ flies (Fig. 3d). This suggests that the dysplasia we observed was the cumulative effect of disrupted ISC or enteroblast differentiation, arising as a non-cell-autonomous effect of decreased autophagy in neighboring ECs, rather than a consequence of increased ISC proliferation. RNAi against *Atg5* in ECs significantly decreased lifespan in male flies, but had no effect in females (Fig. 3e). These data reveal the dimorphic regulation of autophagy in ECs and its impact on gut pathology and lifespan; females have low basal levels autophagy that increase in response to rapamycin treatment, with a consequent reduction in gut pathology and increase in lifespan, whereas males with high basal autophagy see an increase in gut pathology and a reduction in lifespan upon its suppression.

### Ablation of autophagy through the histone–Bchs axis in ECs is sufficient to block lifespan extension in females upon rapamycin and spermidine treatment

**Fig. 4:**
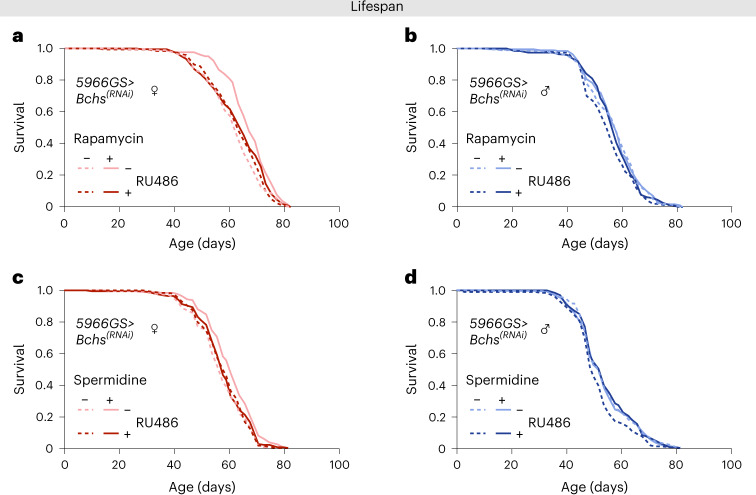
Bchs is a required target for autophagy activation, lifespan extension and intestinal homeostasis from the mTORC1–histone axis. **a**, Knockdown of *Bchs* in ECs of adult females had no effect on lifespan, but it abolished the increase in lifespan in response to rapamycin (long-rank test, control versus RU486 *P* = 0.40, rapamycin versus rapamycin+RU486 *P* = 0.0065, *n* = 198–199 flies per condition). **b**, Knockdown of *Bchs* in ECs of adult males shortened lifespan (long-rank test, control versus RU486 *P* = 0.0095, *n* = 198-200 flies per condition). **c**, Knockdown of *Bchs* in ECs of adult females had no effect on lifespan but abolished the increase in lifespan in response to spermidine (long-rank test, control versus RU486 *P* = 0.54, spermidine versus spermidine + RU486 *P* = 0.012, *n* = 198–199 flies per condition). **d**, Knockdown of *Bchs* in enterocytes of adult males shortened lifespan (long-rank test, control versus RU486 *P* = 0.023, *n* = 199 flies per condition). See also Supplementary Tables 5 and 6. Source data

**Extended Data Fig. 6:**
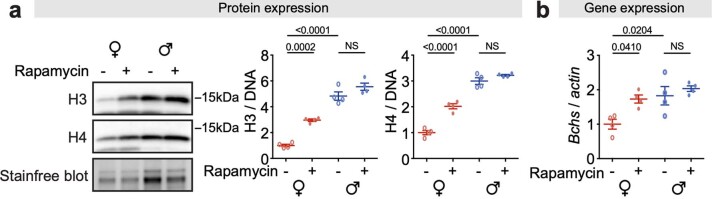
Rapamycin treatment increases expression of histone H3, histone H4 and *Bchs* in intestines of females but not in males. **a**, The expression of histones H3 and H4 in intestines of *w*^*Dah*^ females was lower than in males, and rapamycin treatment (200 µM) increased it, at 10 days of age (n = 4 biological replicates of 10 intestines per replicate, two-way ANOVA, H3, interaction p < 0.05, H4, interaction p < 0.001; post-hoc test). **b**, The expression of *Bchs* in intestines of *w*^*Dah*^ females was lower than in males, and rapamycin treatment (200 µM) increased it, at 10 days of age (n = 4 biological replicates of 10 intestines per replicate, two-way ANOVA, interaction p > 0.05; post-hoc test). Data are presented as mean values ± s.e.m. Source data

Increased intestinal autophagy in response to rapamycin can be mediated through a histones–Bchs axis, where levels of H3 and H4 histone proteins regulate the autophagy cargo adapter *bluecheese* (*Bchs*) in ECs^30^. Publicly available expression data (*FlyAtlas 2*) indicate that *Bchs* is expressed at higher levels in intestines of males than of females^48^. We confirmed that *Bchs* transcript levels, and expression of histone H3 and H4 proteins, were higher in intestines of males compared to females. Rapamycin treatment did not increase either *Bchs* or histone expression further in males but did so in females, to levels comparable with those in males in the case of *Bchs* (Extended Data Fig. 6a,b). To test whether the histone-Bchs axis was required for rapamycin-mediated lifespan extension in females and males, we expressed RNAi against *Bchs* in adult ECs, *5966GS* > *Bchs*^*[RNAi]*^. In line with previous data^30^, knockdown of *Bchs* alone had no effect on lifespan in females, but it blocked lifespan extension upon rapamycin treatment (Fig. 4a). In males, knockdown of *Bchs* shortened lifespan (Fig. 4b), suggesting that the sexually dimorphic level of *Bchs* in ECs mediates the lifespan response to rapamycin treatment. (Fig. 4b).

Spermidine ameliorates age-related functional decline and promotes lifespan in *Drosophila* and mice through activation of autophagy^49,50^. In line with previous finding^50^, we observed female flies had greater lifespan extension in response to spermidine than did in males (Fig. 4c,d). Knockdown of *Bchs* in females blocked lifespan extension upon spermidine treatment (Fig. 4c), whereas knockdown of *Bchs* was sufficient to shorten lifespan in males (Fig. 4d). Together, our results suggest the histone–Bchs axis plays a key role in sexually dimorphic responses to mTOR-autophagy interventions.

### Cellular and molecular responses to TOR-attenuation depend on cell-autonomous sexual identity of ECs

In *Drosophila*, sexual identity of somatic cells is determined in a cell-autonomous manner via the sex determination pathway^51^. Genetic manipulation of the pathway at the level of the splicing factor *transformer* allows for the generation of tissue-specific sexual chimeras^17,52^. We switched sex solely in ECs of males and females using the EC-specific driver *mex1-Gal4*^52–54^ to express or abrogate *transformer*^*Female*^ (*tra*^*F*^).

**Extended Data Fig. 7:**
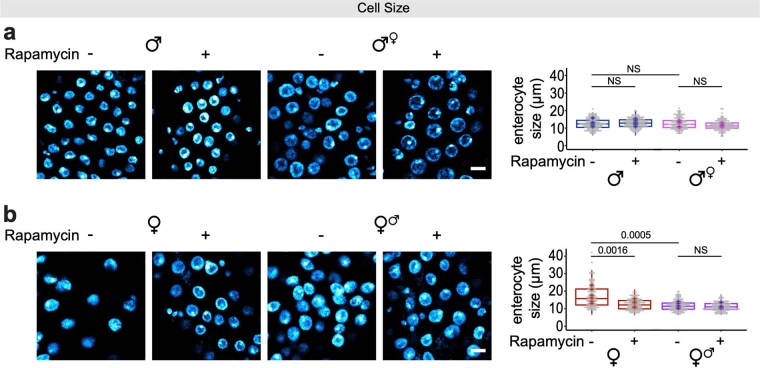
Expression of *tra*^*F*^ is necessary but not sufficient for the larger enterocyte size in female intestines. **a**, Expression of *tra*^*F*^ in ECs in males did not affect cell size, neither did treatment with rapamycin (200 µM), at 20 days of age (control male *mexG4* > *+ vs* feminised male *mexG4* > *tra*^*F*^; scale bar = 10 µm; n = 6-8 intestines, n = 20-25 enterocytes per intestine, circles indicate individual values and diamonds represent the average value per intestine; linear mixed model, interaction p > 0.05; post-hoc test). **b**, Knock-down of *tra*^*F*^ in ECs in females reduced the size of enterocytes to the level of rapamycin-treated females, which was not further reduced by rapamycin treatment (200 µM), at 20 days of age (control female *mexG4* > *+ vs* masculinized female *mexG4* > *tra*^*F [RNAi]*^; scale bar = 10 µm; n = 6-8 intestines, n = 17-23 enterocytes per intestine, circles indicate individual values and diamonds represent the average value per intestine; linear mixed model, interaction p < 0.01; post-hoc test). For box-and-whiskers plots, Median, 25th and 75th percentiles, and Tukey whiskers are indicated. Source data

EC size is regulated both by sex and mTOR-signaling (Fig. 2c). Masculinization of female cells through EC-specific expression of *tra*^*F[RNAi]*^ reduced cell size to that of males, and this effect was not reduced further by treatment with rapamycin (Extended Data Fig. 7b). In contrast, feminization of male ECs by expression of *tra*^*F*^ did not affect their size, and neither did treatment with rapamycin (Extended Data Fig. 7a). This finding suggests that expression of *tra*^*F*^ is necessary, but not sufficient, for the larger cell size observed in female intestines.

**Fig. 5:**
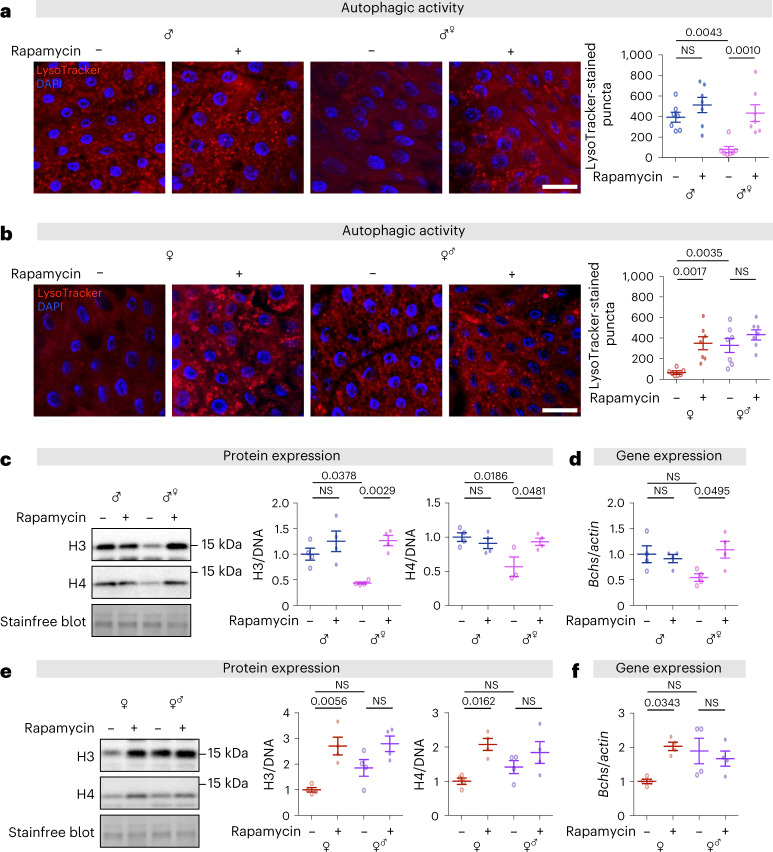
Cell-autonomous sexual identity in enterocytes dictates the levels of autophagy, histones and *Bchs* expressions in response to rapamycin treatment. **a**, Feminization of male guts by expression of *tra*^*F*^ in ECs reduced the number of LysoTracker-stained puncta in the gut, and it restored the response to rapamycin treatment (200 µM) at 10 days of age (control male *mexG4* > *+ vs* feminized male *mexG4* > *tra*^*F*^; scale bar = 20 µm; *n* = 7 intestines per condition; *n* = 2–3 pictures per intestine, data points represent the average value per intestine; linear mixed model, interaction *P* < 0.05; post-hoc test). **b**, Masculinization of female guts by knockdown of *tra*^*F*^ in ECs increased the number of LysoTracker-stained puncta in the gut, and abolished the response to rapamycin treatment (200 µM), at 10 days of age (control female *mexG4* > *+ vs* masculinized female *mexG4* > *tra*^*F [RNAi]*^; scale bar = 20 µm; *n* = 7 intestines per condition; *n* = 2–3 pictures per intestine, data points represent the average value per intestine; linear mixed model, interaction *P* < 0.05; post-hoc test). **c**, Expression of histones H3 and H4 in the gut of feminized males was lower than in males, and rapamycin treatment (200 µM) increased it to the level in males, at 10 days of age (*n* = 3–4 biological replicates of 10 intestines per replicate, two-way ANOVA, H3 and H4, interaction *P* < 0.05; post-hoc test). **d**, Expression of *Bchs* in the gut of feminized males did not significantly lower than in males, whereas rapamycin treatment (200 µM) increased it to the level in males, at 10 days of age (*n* = 4 biological replicates of 10 intestines per replicate, two-way ANOVA, interaction *P* < 0.05; post-hoc test). **e**,**f**, Expression of histones H3, H4 and *Bchs* in the gut of masculinized females did not differ significantly from that in females, and we did not detect an increase upon rapamycin treatment (200 µM), at 10 days of age (*n* = 4 biological replicates of 10 intestines per replicate, two-way ANOVA, H3 and H4, interaction *P* > 0.05; *Bchs*, interaction *P* < 0.05; post-hoc test). Data are presented as mean values ± s.e.m. Source data

Males expressing *tra*^*F*^ in ECs (*mex1-Gal4;UAS-tra*^*F*^) had suppressed basal autophagy in the intestine, which showed a significant increase upon treatment with rapamycin (Fig. 5a), similar to control females. Concordantly, females expressing *tra*^*F[RNAi]*^ in ECs (*mex1-Gal4;UAS-tra*^*F[RNAi]*^) had increased autophagy compared to control females but did not respond to treatment with rapamycin (Fig. 5b), similar to control males. Expression of H3, H4 and *Bchs* was correlated with the level of autophagy in the intestines of sexual chimeras. Feminized males showed a low level of H3, H4 and *Bchs*, which was increased to the same level as that of control males in response to rapamycin treatment (Fig. 5c,d). Masculinized females had similar basal levels of H3, H4 and *Bchs* to control females, and we did not detect an increase response to rapamycin treatment (Fig. 5e,f). Altogether, these data suggest that levels of autophagy in enterocytes are determined cell autonomously by *tra*^*F*^ and that the histone H3/H4-*Bchs* axis plays a key role in regulating sexual dimorphism of intestinal autophagy.

### Sexual identity of ECs influences fecundity and determines the response of intestinal homeostasis and lifespan to rapamycin

**Fig. 6:**
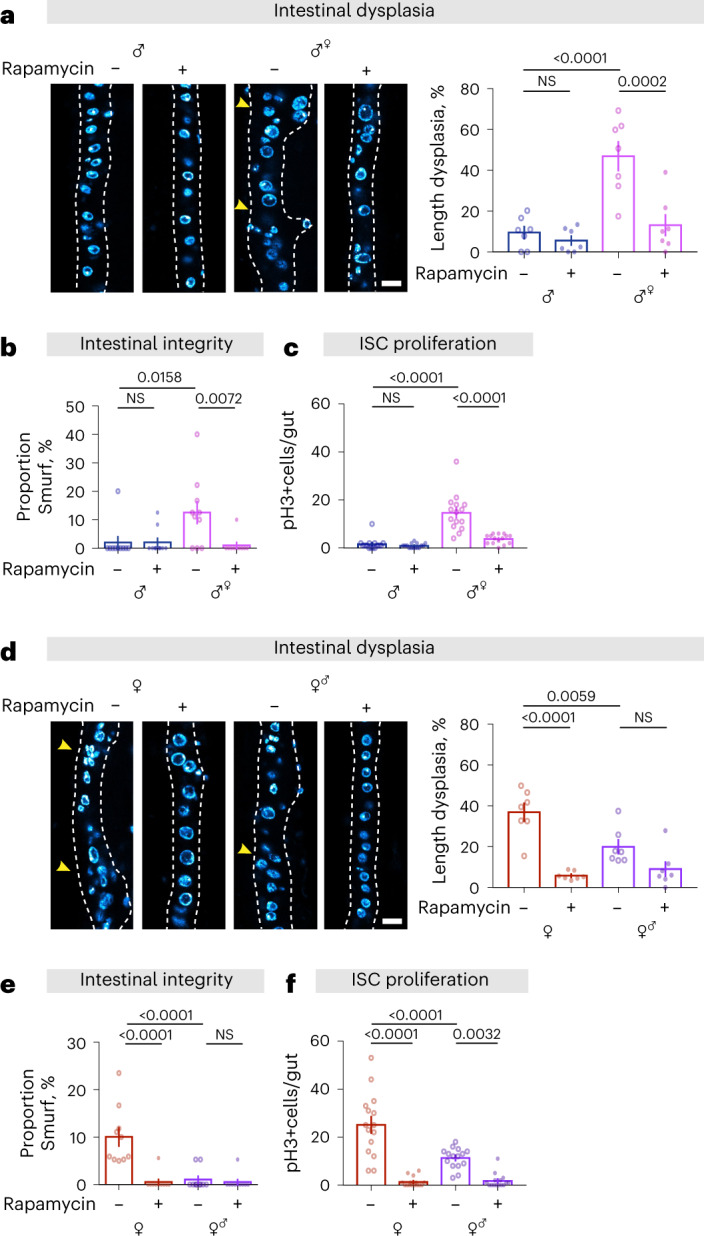
Cell-autonomous sexual identity in enterocytes mediates age-related gut pathology, barrier function and ISC mitoses in response to rapamycin treatment. **a**, Feminization of male guts by expression of *tra*^*F*^ in ECs increased intestinal dysplasia, which was attenuated by rapamycin treatment (200 µM), at 50 days of age (control male *mexG4* > *+ vs* feminized male *mexG4* > *tra*^*F*^; scale bar = 15 µm; *n* = 7 intestines per condition; two-way ANOVA, interaction *P* < 0.01; post-hoc test). **b**, Feminization of male guts by expression of *tra*^*F*^ in ECs increased gut leakiness (number of Smurfs), which was attenuated by rapamycin treatment (200 µM), at 60 days of age. Bar charts show with *n* = 10 biological replicates of 6–12 flies per replicate (two-way ANOVA, interaction *P* < 0.05; post-hoc test). **c**, Feminization of male guts by expression of *tra*^*F*^ in ECs increased the number of pH3^+^ cells, which was attenuated by rapamycin treatment (200 µM), at 20 days of age (*n* = 15 intestines per condition; two-way ANOVA, interaction *P* < 0.001; post-hoc test). **d**, Masculinization of female guts by knockdown of *tra*^*F*^ in ECs decreased intestinal dysplasia, with no further decrease when combined with rapamycin treatment (200 µM), at 50 days of age (control female *mexG4* > *+ vs* masculinized female *mexG4* > *tra*^*F [RNAi]*^; scale bar = 15 µm; *n* = 7 intestines per condition; two-way ANOVA, interaction *P* < 0.01; post-hoc test). **e**, Masculinization of female guts by knockdown of *tra*^*F*^ in ECs decreased gut leakiness, which was not further decreased by the combination of rapamycin treatment (200 µM), at 60 days of age. Bar charts show with *n* = 10 biological replicates of 1,520 flies per replicate (two-way ANOVA, interaction *P* < 0.001; post-hoc test). **f**, Masculinization of female guts by knockdown of *tra*^*F*^ in ECs decreased the number of pH3+ cells, which was further decreased by combination with rapamycin treatment (200 µM), at 20 days of age (*n* = 15 intestines per condition; two-way ANOVA, interaction *P* < 0.001; post-hoc test). Data are presented as mean values ± s.e.m. Source data

Limited cell growth and increased autophagy are correlated with better intestinal homeostasis during ageing in males compared to females (Fig. 2c–e). To determine if this correlation held in individuals with sex-switched ECs, we measured intestinal dysplasia, barrier function and ISC mitosis. In concordance with analyses of autophagy in young individuals, intestinal dysplasia and barrier function were correlated with EC rather than organismal sex, as were the responses of these pathologies to rapamycin (Fig. 6a,b,d,e). ISC mitoses were also affected by EC sex, such that males with feminized ECs had higher numbers of mitoses than controls, whereas females with masculinized ECs had fewer (Fig. 6c,f). These findings are in line with other evidence of non-cell-autonomous effects of EC homeostasis on ISCs^55^.

**Fig. 7:**
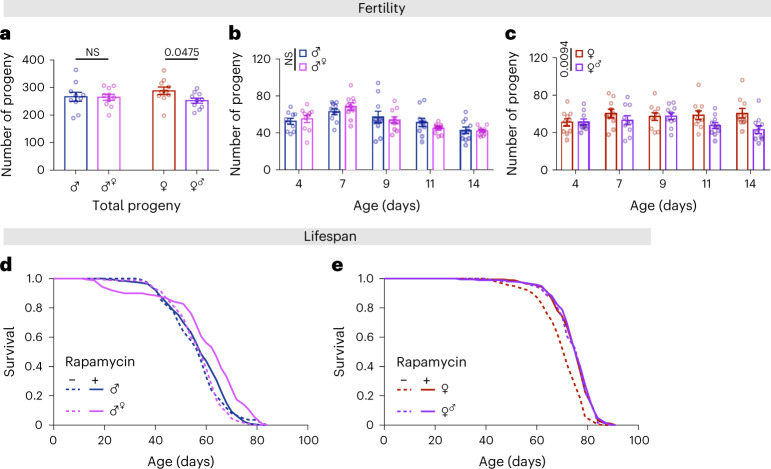
Cell-autonomous sexual identity in enterocytes influences fertility, and it mediates extension of lifespan in response to rapamycin treatment. **a**–**c**, Feminization of male guts by expression of *tra*^*F*^ in ECs did not significantly affect the number of progeny, whereas masculinization of female guts by knock-down of *tra*^*F*^ in ECs reduced the number of progeny (control male *mexG4* > *+* *vs* feminized male *mexG4* > *tra*^*F*^, control female *mexG4* > *+ vs* masculinized female *mexG4* > *tra*^*F [RNAi]*^; *n* = 10 biological replicates of 3 males and 3 females per replicate; two-tailed Student’s *t-*test, NS *P* > 0.05, *P < 0.05 (**a**); or two-way ANOVA, treatment *P* > 0.05 (**b**); two-way ANOVA, treatment *P* < 0.01 (**c**). **d**, Feminization of male guts by expression of *tra*^*F*^ in ECs extended lifespan in response to rapamycin treatment (200 µM) (log-rank test, *P* = 1.55 × 10^−6^, *mexG4* > *tra*^*F*^ control versus *mexG4* > *tra*^*F*^ Rapamycin, *n* = 198–199 flies per condition). See also Supplementary Table 7. **e**, Masculinization of female guts by knock-down of *tra*^*F*^ in ECs extended lifespan, which was not further extend by rapamycin treatment (200 µM) (log-rank test, *P* = 1.56 × 10^−9^ *mexG4* > *+* control versus *mexG4* > *tra*^*F [RNAi]*^ control, *n* = 199 flies per condition). See also Supplementary Table 8. Data are presented as mean values ± s.e.m. Source data

**Extended Data Fig. 8:**
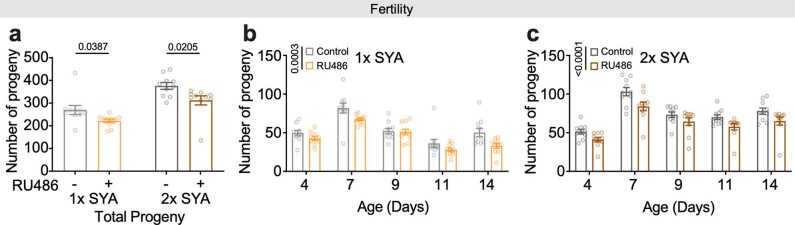
Over-expression of histones in enterocytes reduces fertility. **a-c**, Over-expression of Histones H3/H4 in adult ECs in females (*5966GS* > *H3/H4*) reduced the number of progeny, both in flies fed control food (1x SYA) and those fed food with a doubled yeast content (2x SYA) (n = 10 biological replicates of 3 males and 3 females per replicate, (**a**) students t test, *p < 0.05; (**b,c**) two-way ANOVA, treatment p < 0.001). Data are presented as mean values ± s.e.m. Source data

Gut growth via ISC division^52,56^, and some aspects of intestinal metabolism^57^, affect fertility in females and males, respectively. To determine whether enterocyte sex can influence reproductive output, we measured fertility in individuals with sex-switched ECs. We did not detect a difference in the fertility of EC-feminized males compared to that of control males (Fig. 7a,b). However, EC-masculinized females showed moderately, but significantly, decreased fertility compared to that of control females (Fig. 7a,c). To understand whether this is mediated by the H3/H4-autophagy axis, we assessed fertility in females with increased H3/H4 expression in ECs, which we have previously demonstrated have an increased lifespan as a consequence of increased EC autophagy^30^. We assessed this on two levels of yeast to understand whether increased autophagy limits reproduction under specific nutritional conditions. We observed a small but significant reduction of fertility in enterocyte H3/H4-overexpressing females, both in flies fed control food and those fed food with doubled yeast (Extended Data Fig. 8a–c).

Feminized males showed a lifespan extension upon treatment with rapamycin that was not observed in control males (Fig. 7d). In contrast, masculinized females did not have extended lifespan in response to rapamycin (Fig.7e). Interestingly, the lifespan of gut-masculinized females on both rapamycin-treated and control food was comparable to that of control females treated with rapamycin (Fig. 7e). Taken together, these results suggest that the intrinsic sexual identity of ECs determines the effect of rapamycin on intestinal homeostasis and lifespan, regardless of organismal sex.

### Sexually dimorphic responses to rapamycin are conserved in mice

**Fig. 8:**
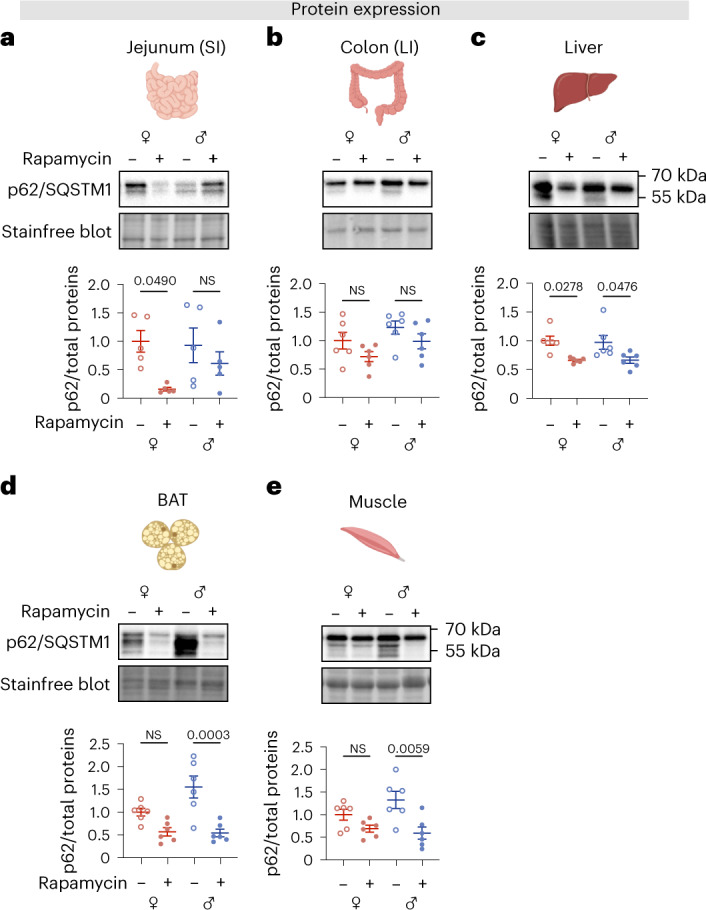
Sex differences in basal autophagy levels and responses to rapamycin are detected in mouse tissues. **a**–**e**, The expression of p62/SQSTM1 in the jejunum (small intestine (SI)), colon (large intestine (LI)), liver, BAT and muscle of female and male mice. **a**, Rapamycin induced a significant reduction of p62/SQSTM1 protein level in jejunums in females that was not detected in males (*n* = 5 biological replicates of one mouse per replicate, two-way ANOVA, treatment *P* < 0.05, sex *P* = 0.37, interaction *P* = 0.23, post-hoc test). **b**, Higher basal level of p62/SQSTM1 in males detected by two-way ANOVA, whereas rapamycin induced similar reductions in p62/SQSTM1 in the two sexes (*n* = 6 biological replicates of one mouse per replicate, two-way ANOVA, treatment *P* < 0.05, sex *P* < 0.05, interaction *P* = 0.81, post-hoc test). **c**, Rapamycin markedly reduced p62/SQSTM1 protein level in the liver of both sexes (n = 6 biological replicates of one mouse per replicate, two-way ANOVA, treatment *P* < 0.001, sex *P* = 0.87, interaction *P* = 0.86, post-hoc test). **d**,**e**, Rapamycin significantly reduced p62/SQSTM1 protein level in the BAT and muscle of males (*n* = 6 biological replicates of one mouse per replicate, two-way ANOVA, BAT: treatment *P* < 0.0001, sex *P* = 0.08, interaction *P* = 0.05, post-hoc test; muscle: treatment *P* < 0.01, sex *P* = 0.41, interaction *P* = 0.14, post-hoc test). All mice were sacrificed and tissues were collected at 12 months of age. Data are presented as mean values ± s.e.m. Source data

**Extended Data Fig. 9:**
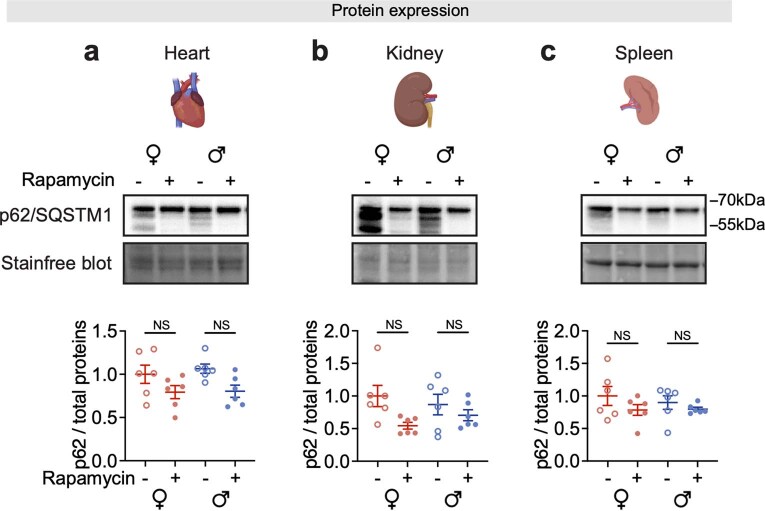
Dimorphic responses to rapamycin in mice tissues. **a-c**, The expression of p62/SQSTM1 in the heart, kidney, and spleen of female and male mice (n = 6 biological replicates of one mouse per replicate, two-way ANOVA, Heart: treatment p < 0.01, sex p = 0.64, interaction p = 0.74, post-hoc test; Kidney: treatment p < 0.05, sex p = 0.91, interaction p = 0.25, post-hoc test; Spleen: treatment p = 0.13, sex p = 0.66, interaction p = 0.57, post-hoc test). All mice were sacrificed and tissues were collected at 12 months of age. Data are presented as mean values ± s.e.m. Source data

To test whether the interactions among sex, autophagy and rapamycin that we observed in *Drosophila* were conserved in mice, we assessed levels of autophagy in mouse tissues. Decreased levels of p62/SQSTM1 can be observed when autophagy is induced in mice^58^, and we measured its levels in a range of tissues collected from control and rapamycin-fed female and male mice at 12 months of age. (Fig. 8a–e and Extended Data Fig. 9a-c). Rapamycin treatment significantly reduced the level of p62/SQSTM1 in the jejunum, colon, liver, brown adipose tissue (BAT), muscle (Fig. 8a–e), heart and kidney, but not spleen (Extended Data Fig. 9a–c), indicating an increase in autophagy in most, but not all, tissues in response to rapamycin treatment. In four out of these eight tissues we detected sex differences, either in basal autophagy levels or in the response to rapamycin. Notably, we detected a significantly increased autophagy signature in response to rapamycin in the jejunum of the small intestine (SI) in female mice, which was not present in males (Fig. 8a). In the colon, although post-hoc testing did not find a significant effect of rapamycin in either sex, ANOVA detected an effect of both sex and treatment on autophagy levels (Fig. 8b). Conversely, we detected significantly increased autophagy in response to rapamycin in BAT and skeletal muscle from male, but not female, mice, possibly attributable to a higher baseline of p62/SQSTM1 protein level in males, which reduced to a level comparable to that of females upon treatment (Fig. 8d,e). Altogether, we find that autophagic responses to rapamycin are tissue specific and can be sexually dimorphic in mice, including in the intestine.

## Discussion

The IIS/mTOR signaling network regulates dimorphic, complex traits such as metabolism, growth and lifespan^23,59–61^. However, it is not well understood how dimorphisms in IIS/mTOR-regulated traits affect tissue aging and responses to geroprotective drugs. *Drosophila* females treated with rapamycin show a strong lifespan extension in response to treatment with rapamycin^28^, and the fly offers a tractable system for understanding dimorphisms in tissue ageing^17^ and responses to anti-aging therapeutics^20,62^. Treatment of *Drosophila* with rapamycin extended lifespan in females, but not in males, regardless of their genetic background. Rapamycin increased autophagy and reduced cell size of intestinal ECs in females. We found a striking dimorphism in basal metabolism of ECs; in males, autophagy was constitutively high, cell size was smaller than in females and both autophagy and cell size were insensitive to mTORC1 attenuation by rapamycin. This raises the possibility that intestinal autophagy is actively buffered in males or is maintained at an upper limit by constraints on the availability of autophagy components in ECs. One consequence of increased intestinal autophagy in males was attenuated age-related intestinal barrier function decline, underpinning the overall slower progression of age-related intestinal pathologies in males compared to females. Intestinal barrier function maintenance, independent of ISC division, is a key determinant of lifespan in *Drosophila*. This effect has been demonstrated in multiple ways in females through manipulation of diet^63^ or the microbiome^38^ and through genetic targeting of junctional components^64^ or upstream signaling pathways^30,65^. Males do not usually respond strongly to manipulations that attenuate functional decline of the intestine^17,62^, including rapamycin^28^ (this study), probably because progression of intestinal pathology is slow. Here, we showed that males were also sensitive to barrier function decline by genetically targeting autophagy components, which increased the incidence of barrier function failure and decreased lifespan.

A specific autophagy pathway, regulated by histones H3/H4 and requiring the cargo adapter Bchs/WDFY3, maintains junctional integrity in ECs in the intestine in females during aging^30^. Autophagy in ECs also lowers sensitivity to reactive oxygen species induced by commensal bacteria, via suppression of p62 and Hippo pathway genes, to maintain septate junction integrity and attenuate dysplasia^66^. Maintenance of cell junctions by increased autophagy is not restricted to epithelial tissue; for example, this increase occurs acutely in mammalian endothelial cells to prevent excessive diapedesis of neutrophils in inflammatory responses^67^. We found a link between EC sex, the histone–Bchs axis, junctional integrity, and lifespan. We showed that the histone-Bchs axis acts as a regulator to mediate autophagy-dependent longevity interventions, such as rapamycin and spermidine. Cell-autonomous sexual identity of ECs determined their histone and *Bchs* levels, and subsequently their basal level of autophagy. Autophagy is key to maintaining junctional integrity in ECs and, consequently, barrier function of the intestine. Thus, the sex-determined metabolic state of ECs, including basal autophagy and cell size, dictates how they respond to rapamycin treatment; at the cellular level, at the level of organ physiology, and at the level of whole organism homeostasis during ageing to influence lifespan^61,68^.

Why do males and females take such different approaches to intestinal homeostasis? Females pay a cost for maintaining their intestine in an anabolic state, with lower autophagy, higher cell growth and higher rates of stem cell division^17,52^ (this study), leading to pathology and dysplasia at older ages^17^. Selection acts weakly on age-related traits and strongly on those promoting fitness in youth^69^, and females require hormone-regulated intestinal cell growth and organ size plasticity to maintain egg production at younger ages^56,70^. We found that metabolic responses of the intestine to mTOR attenuation, including autophagy and cell growth, were regulated by *tra* cell autonomously. Sensitivity to nutrients, particularly protein levels, in the diet is important for females to maintain and regulate egg production^71^, and we found that female ECs had a cell-autonomous sensitivity to changes in mTOR signaling. This sensitivity may be an adaptive mechanism to maintain reproductive output in the face of fluctuating nutrient availability^72^, where females can take advantage of higher protein by resizing ECs^73^, in addition to post-mating organ growth achieved through stem cell division^52,56^. We showed that females with masculinized ECs, which have a smaller cell size and higher autophagy, have reduced fertility. This effect is similar to the reduction in fertility demonstrated when ISCs are masculinized in female guts^52^, suggesting that sex-determination signaling regulates organ size plasticity through both cell growth and cell division. In addition, overexpression of histones H3/H4 in adultECs in females reduced fertility, similar to masculinized ECs, suggesting a key role for histones in dimorphic physiology regulated by sex-determination signaling in flies. Although fertility was reduced, females with masculinized (this study) or histone-overexpressing^30^ ECs had healthier guts over during ageing and a longer lifespan, supporting the idea that in females, early life reproduction trades off with intestinal homeostasis at older ages^70^.

Interestingly, males with feminized ECs did not show an increase in EC cell size, suggesting that *tra*^*F*^ is necessary, but not sufficient, to induce EC growth, contrary to the effect seen on whole-body size when *tra*^*F*^ is expressed throughout the developing larva^74^. Females produce larger ECs when fed with a high-protein diet or through genetically activating mTOR or blocking autophagy by manipulation of mTOR-autophagy cascade core components in a cell-autonomous manner^73^. However, we found that manipulating EC sex, and consequently autophagy levels, did not lead to larger cells in males. Together, these data suggest that feminizing ECs by overexpression of *tra*^*F*^ in male guts does not simply recapitulate autophagy reduction by EC-specific knockdown of *Atg5*. One possibility is that feminized ECs maintain better nutrient absorption during aging, a known determining factor of lifespan^75,76^, counteracting the effect of increased pathology and leading to comparable lifespan to males on control food.

Male fertility was unaffected by feminization of ECs. Male fitness may rely more heavily on nutrients other than yeast-derived protein, particularly carbohydrates, where nonautonomous regulation of sugar metabolism in the male gut by the testis has been shown to be essential for sperm production^57^. The sexes, therefore, rely on distinct metabolic programs to maintain fitness. Cellular growth and size plasticity of the gut may not increase fitness in males, and as a result, they may maintain their intestines at a low catabolic limit that cannot be pushed further by lowered mTOR. Sexually antagonistic traits can be resolved by sex-specific regulation^77^. Direct regulation of cell growth and autophagy (this study) and stem cell activity^52^ by sex-determination genes may allow males and females to diverge in their energetic investment in the gut, and this effect may interact with fertility and pathophysiology, which can eventually determine lifespan.

Targeted mTORC1 inhibition by the drug rapamycin extends lifespan more in female than in male mice^25,78^. Although there is evidence that off-target effects of rapamycin on hepatic mTORC2 signaling via *Rictor* can reduce the lifespan of male mice^79^, dimorphic effects of rapamycin treatment on lifespan may also be regulated by other, complex interactions with specific tissues and through interaction with environmental factors such as the microbiome^27^. Responses of lifespan to rapamycin treatment in mice were dose dependent, and we do not yet know the maximum lifespan extension that can be achieved, in either sex, through chronic treatment with the drug. In one study, female mice were found to have higher circulating levels of rapamycin than did males for a given dose in the food^25^, suggesting that sex differences in drug metabolism or bioavailability could play a role in dimorphic responses to pharmaceutical therapies^13^.

Here, we demonstrate that sex differences in basal levels of autophagy and responses to rapamycin are present in mice, including in the intestine. In this and other studies, there are measurable sex differences in expression of autophagy-related genes (for example, spinal cord and muscle tissue^80^) and autophagy proteins (for example, LC3B in the heart^81^ and p62/SQSTM1 in BAT and skeletal muscle (this study)), pointing to higher basal levels of autophagy across tissues in male mice compared to females. Sex differences in autophagy have been detected from early development and into adulthood in mammals and are speculated to contribute to the greater female vulnerability to age-related disorders such as Alzheimer’s disease^82^. More broadly, sex differences in baseline metabolism may profoundly influence responses to a broad range of treatments for such age-related disorders, particularly those that target nutrient-sensing pathways.

Understanding sex differential responses to geroprotective interventions gives an understanding of the mechanistic underpinnings of sex differences in the intrinsic rate of aging in specific tissues^15,83^, including sex-specific tradeoffs. When we treat age-related disease, we are not treating individuals with equal case histories; instead, we are treating individuals impacted by a lifetime of differences, including those regulated by sex. Understanding conserved mechanisms regulating dimorphism and determining responses to therapeutics will facilitate the development of personalized treatments.

## Method

### Statement

Our research complies with all relevant ethical regulations. Mouse experiments were performed in accordance with the recommendations and guidelines of the Federation of the European Laboratory Animal Science Association, with all protocols approved by the Landesamt für Natur, Umwelt und Verbraucherschutz, Nordrhein-Westfalen, Germany (reference number 81-02.04.2020.A152).

### Fly stocks and husbandry

All transgenic lines were backcrossed for at least six generations into the outbred line, white Dahomey (*w*^*Dah*^), maintained in population cages (unless specified otherwise in figure legends). Wolbachia-positive males and females were used, unless otherwise stated. Stocks were maintained and experiments conducted at 25 °C on a 12 h/12 h light/dark cycle at 60% humidity, on sugar-yeast-agar food (1× SYA) containing 10 % (w/v) brewer’s yeast, 5% (w/v) sucrose and 1.5% (w/v) agar unless otherwise noted. The following stocks were used in this study: *UAS-Atg5*^*[RNAi]*^^84,85^, *UAS-H3/H4* (this lab)^30^, *UAS-tra*^*F*^ (Bloomington, 4590), *UAS-tra*^*F[RNAi]*^ (Bloomington, 44109), *UAS-Bchs*^*[RNAi]*^ (Vienna, KK110785), *mex1-Gal4* (Bloomington, 91369), *5966GS*^86^, *Dah*^87^, *DGRP-OX*^33^.

### Lifespan assay

Files were reared at standard density before being used for lifespan experiments. Crosses were set up in cages with grape juice agar plate. The embryos were collected in PBS and squirted into bottles at 20 µl per bottle to achieve standard density. The flies were collected over a 24 h period and allowed 48 h to mate after eclosing as adults. Flies were subsequently lightly anaesthetized with CO_2_, the adults were sorted into the vials at a density of 20 per vial. For lifespans with rapamycin (50 µM, 200 µM and 400 µM) (LC Laboratories) and/or RU486 (100 µM) (Sigma-Aldrich), drugs were dissolved in ethanol and added to food. For lifespans with spermidine (1 mM) (Sigma-Aldrich), drug was dissolved in distilled H_2_O and added to food.

### Fertility assay

All fertility assays were performed on vials housing 3 virgin females and 3 virgin males that were all 2 days old. All assays were performed on 10 replicates per group. Flies were transferred to new vials every 2–3 days, and flies were discarded after the fifth ‘flip’. To assess overall fertility, we counted emergence of pupal progeny, as previously described^88^.

### Gut leakiness assay (Smurf assay)

Flies were aged on normal 1× SYA food and then switched to SYA food containing 2.5% (w/v) Brilliant blue FCF (Sigma-Aldrich). Flies were examined after 48 h, as previously described^17,35^.

### Mouse husbandry

C3B6F1 hybrid mice were generated by a cross between C3H female and C57BL/6 J male mice from our in-house animal facility. C3H and C57BL/6 J mice were originally from Charles River Laboratories. Whereas females were randomized upon weaning, male mice were weaned litterwise to avoid aggression and fighting. All mice were housed in individually ventilated cages, in groups of five mice per cage, under specific-pathogen-free conditions, at 21 °C, with 12 h light/dark cycle and 50-60% humidity. Mice received a standard rodent diet (Ssniff Spezialdiäten; 9% fat, 34% protein and 57% carbohydrates) and drinking water at all times. The rapamycin treatment group received rapamycin (42 mg kg^−1^ body weight, microencapsulated in Eudragit S100) from 3 months of age when the control group received Eudragit encapsulation medium only. Mice were fasted for 18 h before euthanasia and tissues were collected, at 12 months of age.

### Immunoblotting

Fly and mouse tissues were homogenized in 80 µl 1× RIPA Lysis and Extraction Buffer (Thermo Fisher Scientific) containing PhosSTOP (Roche) and cOmplete, Mini, EDTA-free Protease Inhibitor Cocktail (Roche), except for fly guts which were homogenized in 80 µl self-prepared trichloroacetic acid lysis extraction buffer. Extracts were then cleared by centrifugation, protein content determined by using Pierce BCA Protein Assay (Thermo Fisher Scientific) and DNA content determined by using Qubit dsDNA HS Assay Kit (Invitrogen) on a Qubit 3.0 Fluorometer (Thermo Fisher Scientific). Approximately 8 µg protein extract was loaded per lane on polyacrylamide gel (4-20% Criterion, BioRad). Proteins were separated and transferred to polyvinylidene difluoride membrane. Following antibodies were used: Atg8a (homemade, gift from P. Nagy’s lab, Eötvös Loránd University, Hungary, 1:5,000), phospho-*Drosophila*p70 S6 kinase (Thr398) (Cell Signaling, 9209, 1:1,000), total S6K (homemade from this lab, 1:1,000), histone H3 (Abcam, ab1791, 1:10,000), histone H4 (Active Motif, 39269, 1:3,000) and p62/SQSTM1 (Abcam, ab56416, 1:1,000). Horseradish peroxidase (HRP)-conjugated secondary antibodies goat anti-rabbit IgG antibody, HRP conjugate (Sigma-Aldrich, 12-348, 1:10,000) and Goat Anti-Mouse IgG Antibody, HRP-conjugate (Sigma-Aldrich, 12-349, 1:10,000) were used. Blots were developed using the ECL detection system (Amersham). Immunoblots were analyzed using Image Lab (v5.1, Bio-Rad).

### RNA isolation and quantitative RT-PCR

Tissue was dissected, frozen on dry ice and stored at −80 °C. Total RNA from guts of 10 flies was extracted using TRIzol (Invitrogen) according to the manufacturer’s instructions. mRNA was reverse transcribed using random hexamers and the SuperScript III First Strand system (Invitrogen). Quantitative PCR was performed using Power SYBR Green PCR (Applied Biosystems) on a QuantStudio 6 Flex System with QuantStudio Real-time PCR software (v.1.7.1, Applied Biosystems) by following the manufacturer’s instructions. Primers for quantitative RT-PCR included Bchs_F1: 5′-AGCCTCACCACGCTAAAGAAG-3′; Bchs_R1: 5′-CTCATGTCGTTTGACGGACAG-3′; Act5C_F1: 5′-AGGCCAACCGTGAGAAGATG-3′; Act5C_R1: 5′-GGGGAAGGGCATAACCCTC-3′.

### LysoTracker and Cyto-ID staining, imaging and image analysis

LysoTracker dye accumulates in low-pH vacuoles, including lysosomes and autolysomes, and Cyto-ID staining selectively labels autophagic vacuoles. Combination of both gives a better assessment of entire autophagic process^30,43^. For the dual staining, complete guts were dissected in PBS and stained with Cyto-ID (Enzo Life Sciences, 1:1,000) for 30 min and then with LysoTracker Red DND-99 (Thermo Fisher Scientific, 1:2,000) and Hoechst 33342 (1 mg ml^−1^, 1:1,000) for 3 min. For the experiment only with LysoTracker staining, guts were stained with LysoTracker Red and Hoechst 33342 directly after dissection. Guts were mounted in Vectashield (Vector Laboratories, H-1000) immediately. Imaging was performed immediately using a Leica TCS SP8 confocal microscope with a ×20 objective plus ×5 digital zoom in and Leica Application Suite X (LAS X, Leica). Three separate images were obtained from each gut. Settings were kept constant between the images. Images were analyzed by Imaris (v9.1, Oxford Instruments). This experiment was carried out under blinded conditions.

### Immunohistochemistry and imaging of the *Drosophila* intestine

The following antibodies were used for immunohistochemistry of fly guts: primary antibody, phospho-histone H3 (Ser10) (Cell Signaling, 9701, 1:200); secondary antibody, Alexa Fluor 594 goat anti-rabbit (Thermo Fisher Scientific, A11012, 1:1,000). Guts were dissected in PBS and immediately fixed in 4% formaldehyde for 30 min and subsequently washed in 0.1% Triton-X/PBS (PBST), blocked in 5% BSA / PBST, incubated in primary antibody overnight at 4 °C and in secondary antibody for 1 h at room temperature. Guts were mounted in Vectashield, scored and imaged as described above. For dysplasia measurement, the percentage intestinal length was blind-scored from luminal sections of the R2 region of intestines. For gut cell size measurement, nearest-neighbor internuclear distance in the R2 region was measured from raw image flies using the measure function in Fiji (v2.1.0, ImageJ) (20 distances per gut, *n* ≥ 6 guts per condition). This experiment was carried out under blinded conditions.

### Library preparation and 16 S sequencing/data analysis

Flies were washed in ethanol, and then midguts were dissected in single PBS droplets and 20 guts pooled per replicate. DNA extraction was performed using the DNeasy Blood&Tissue Kit (Qiagen) following the manufacturer’s instructions for gram-positive bacterial DNA and using 0.1 mm glass beads and a bead beater for 45 s at 30 Hz. Library preparation was performed following Illumina’s 16 S Metagenomic Sequencing Library Preparation guide, with the following alterations: 100 ng initial DNA amount, reactions for V3-V4 primer pair, amplicon clean-up with GeneRead Size Selection Kit following the DNA library protocol and BstZ17I digest + gel extraction between PCR reactions for V3-V4 amplicons (for Wolbachia sequence removal). Pooled libraries were sequenced to 100,000 reads/sample on a HiSeq 2x250 bp. Analysis was performed after quality control and paired-end joining for V3-V4 using the Qiime 1 pipeline and the greengenes database, at a depth of 22,000 reads/sample. Remaining *Wolbachia* sequences were removed bioinformatically before further analysis. For total quantification, qPCR with V3-V4 primers was performed with extension time of 1 min. For validation, *A. pomorum* absolute amount was quantified by qPCR using bacteria-specific primers.

### Statistics and reproducibility

Statistical analyses were performed in Prism (v7.0, Graphpad) or R studio (R v3.5.5), except for the log-rank test, which was performed using Excel 2016 (Microsoft). No statistical method was used to predetermine sample size, but we used similar sample sizes as our previous publications^17,30,89^. No specific methods were used to randomly allocate samples to groups. Data collection and analysis were carried out in an unblinded fashion unless otherwise stated. No data were excluded from the analysis. Sample sizes and statistical tests used are indicated in the figure legends, and a Tukey post-hoc test was applied to multiple comparisons correction. Data distribution was assumed to be normal, but this was not formally tested. Error bars are shown as s.e.m. For box-and-whiskers plots, median, 25th and 75th percentiles, and Tukey whiskers are indicated.

### Reporting summary

Further information on research design is available in the Nature Portfolio Reporting Summary linked to this article.

### Supplementary information

Supplementary Information Supplementary Tables 1–8.

Reporting Summary

## Data Availability

The *Drosophila melanogaster* gut microbiota is publicly available at the NCBI BioProject database (PRJNA877614). All other data of this study are available as Source Data files or from the corresponding authors upon reasonable request.

## Source data

Source Data Fig. 1 Numerical Source Data.
Source Data Fig. 1 Uncropped and unprocessed western blot images.
Source Data Fig. 2 Numerical Source Data.
Source Data Fig. 2 uncropped and unprocessed western blot images.
Source Data Fig. 3 Numerical Source Data.
Source Data Fig. 4 Numerical Source Data.
Source Data Fig. 5 Numerical Source Data.
Source Data Fig. 5 uncropped and unprocessed western blot images.
Source Data Fig. 6 Numerical Source Data.
Source Data Fig. 7 Numerical Source Data.
Source Data Fig. 8 Numerical Source Data.
Source Data Fig. 8 uncropped and unprocessed western blot images.
Source Data Extended Data Fig. 1 Numerical Source Data.
Source Data Extended Data Fig. 2 Numerical Source Data.
Source Data Extended Data Fig. 3 Numerical Source Data.
Source Data Extended Data Fig. 3 uncropped and unprocessed WB images.
Source Data Extended Data Fig. 4 Numerical Source Data.
Source Data Extended Data Fig. 5 Numerical Source Data.
Source Data Extended Data Fig. 6 Numerical Source Data.
Source Data Extended Data Fig. 6 uncropped and unprocessed western blot images.
Source Data Extended Data Fig. 7 Numerical Source Data.
Source Data Extended Data Fig. 8 Numerical Source Data.
Source Data Extended Data Fig. 9 Numerical Source Data.
Source Data Extended Data Fig. 9 uncropped and unprocessed western blot images.
