# Supplementary material for: Sexual identity of enterocytes regulates autophagy to determine intestinal health, lifespan and responses to rapamycin
Source: Nat Aging. 2022 Dec 1;2(12):1145–58. doi: 10.1038/s43587-022-00308-7 (PMC10154239; doi:10.1038/s43587-022-00308-7)
Supplement: Supplementary file 1 — Supplementary Tables 1–8. [file 43587_2022_308_MOESM1_ESM.pdf]

nature aging

Article

https://doi.org/10.1038/s43587-022-00308-7

Sexual identity of enterocytes regulates autophagy to determine intestinal health, lifespan and responses to rapamycin

In the format provided by the authors and unedited

Supplementary Information

Supplementary Tables

Supplementary Table 1 - Rapamycin treatment extended lifespan in w^{Dah} females but not in males (related to Figure 1a)

Genotype/Treatment	Median Lifespan (days)	Maximum Lifespan (days)	n Dead	n Censored	% Increase (med) vs control (Control)	% Increase (max) vs control (Control)	Control	p-value (log rank)		
								female - Rapamycin	male - Control	male - Rapamycin
w ♀ - Control	65,5	88	138	5			*	2,07848E-06	1,70202E-24	1,21194E-21
w ♀ - Rapamycin	70,1	88	152	1	7,0%	0,0%			3,21739E-37	2,0062E-34
w ♂ - Control	51,6	78	167	4			*			0,77371499
w ♂ - Rapamycin	51,6	78	153	1	0,0%	0,0%				

Cox Proportional Hazard (CPH) analysis

Details	Coefficient	Coefficient (estimate)	exp(coeff)	SE (coeff)	z	p
dead = 612	Rapamycin	-0,531	0,588	0,123	-4,32	1,60E-05
censored = 11	Sex	1,172	3,230	0,120	9,78	2,00E-16
	Rapamycin:Sex	0,486	1,626	0,166	2,93	3,40E-03

Supplementary Table 2 - Rapamycin treatment extended lifespan in w^{Dah} females but not in males (related to Figure 1b and Extended Data Figure 1)

Genotype/Treatment	Median Lifespan (days)	Maximum Lifespan (days)	n Dead	n Censored	% Increase (med) vs control (Control)	% Increase (max) vs control (Control)	Control	p-value (log rank)		
								w♀ - Rapamycin 50 µM	w♀ - Rapamycin 200 µM	w♀ - Rapamycin 400 µM
w♀ - Control	46	70	118	0			*	9,00928E-08	0,001219441	0,039950423
w♀ - Rapamycin 50 µM	55,1	75	126	1	19,8%	7,1%			0,019868832	8,77649E-08
w♀ - Rapamycin 200 µM	50,4	70	127	2	9,6%	0,0%				0,004560833
w♀ - Rapamycin 400 µM	50,4	70	149	1	9,6%	0,0%				
								w♂ - Rapamycin 50 µM	w♂ - Rapamycin 200 µM	w♂ - Rapamycin 400 µM
w♂ - Control	43,5	68	130	1			*	0,60448407	0,75476739	0,995754772
w♂ - Rapamycin 50 µM	41	68	116	2	-5,7%	0,0%			0,445226822	0,722975386
w♂ - Rapamycin 200 µM	43,5	77	122	1	0,0%	13,2%				0,771149392
w♂ - Rapamycin 400 µM	43,5	66	130	1	0,0%	-2,9%				

Supplementary Table 3 - Rapamycin treatment extended lifespan in *Dah* and *DGRP-OX* females but not in males (related to Extended Data Figure 2a,b)

Dah

Genotype/Treatment	Median Lifespan (days)	Maximum Lifespan (days)	n Dead	n Censored	% Increase (med) vs control (Control)	% Increase (max) vs control (Control)	Control	p-value (log rank) female - Rapamycin	p-value (log rank) male - Control	p-value (log rank) male - Rapamycin
Dah ♀ - Control	85,5		166	32			*	0,039377701	9,71687E-26	3,4958E-23
Dah ♀ - Rapamycin	88		153	40	2,9%				2,52333E-38	5,21998E-35
Dah ♂ - Control	69		168	41			*			0,725633352
Dah ♂ - Rapamycin	71,5		148	40	3,6%					

DGRP-OX

Genotype/Treatment	Median Lifespan (days)	Maximum Lifespan (days)	n Dead	n Censored	% Increase (med) vs control (Control)	% Increase (max) vs control (Control)	Control	p-value (log rank) female - Rapamycin	p-value (log rank) male - Control	p-value (log rank) male - Rapamycin
DGRP ♀ - Control	71		182	7			*	0,016663578	2,14255E-10	8,54343E-06
DGRP ♀ - Rapamycin	74		186	2	4,2%				1,07201E-18	1,62278E-11
DGRP ♂ - Control	64,5		178	4			*			0,234867711
DGRP ♂ - Rapamycin	64,5		174	7	0,0%					

Supplementary Table 4 - Knock-down of Atg5 in ECs shortened the lifespan only in males (related to Figure 3e)

Genotype/Treatment	Median Lifespan (days)	Maximum Lifespan (days)	n Dead	n Censored	% Increase (med) vs control (Control)	% Increase (max) vs control (Control)	Control	p-value (log rank)		
								5966GS>Atg5 [RNAi] ♀ - RU486	5966GS>Atg5 [RNAi] ♂ - Control	5966GS>Atg5 [RNAi] ♂ - RU486
5966GS>Atg5 [RNAi] ♀ - Control	67	85	188	11			*	0,800188548	1,73274E-25	3,60157E-42
5966GS>Atg5 [RNAi] ♀ - RU486	67	81	187	12	0,0%	-4,7%			9,27118E-25	2,19098E-40
5966GS>Atg5 [RNAi] ♂ - Control	56	81	174	25			*			0,004539336
5966GS>Atg5 [RNAi] ♂ - RU486	52	78	192	7	-7,1%	-3,7%				

Cox Proportional Hazard (CPH) analysis

Details	Coefficient	Coefficient (estimate)	exp(coeff)	SE (coeff)	z	p
dead = 741	sex	1,1378	3,1199	0,1097	10,37	0,00000
censored = 55	RU486	0,00491	1,0503	0,1043	0,47	0,638
	RU486:sex	0,3004	1,3503	0,1481	2,03	0,043

Supplementary Table 5 - Knock-down of Bchs in ECs shortened the lifespan only in males (related to Figure 4a,b)

Genotype/Treatment	Median Lifespan (days)	Maximum Lifespan (days)	n Dead	n Censored	% Increase (med) vs control (Control)	% Increase (max) vs control (Control)	Control	p-value (log rank) 5966GS>Bchs [RNAi] ♀- RU486	5966GS>Bchs [RNAi] ♀- Rapamycin	6GS>Bchs [RNAi] ♀- Rapamycin+RU486
5966GS>Bchs [RNAi] ♀- Control	62	77	194	5			*	0,399397332	2,28913E-06	0,068086025
5966GS>Bchs [RNAi] ♀- RU486	62	75	196	3	0,0%	-2,6%			4,10667E-05	0,23414429
5966GS>Bchs [RNAi] ♀- Rapamycin	66	82	185	13	6,5%	6,5%				0,006523441
966GS>Bchs [RNAi] ♀- Rapamycin+RU48	64	81	197	2	3,2%	5,2%				

Cox Proportional Hazard (CPH) analysis

Details	Coefficient	Coefficient (estimate)	exp(coeff)	SE (coeff)	z	p
dead = 795	Rapamycin	-0,45	0,6376	0,1031	-4,36	0,00001
censored = 23	RU486	-0,0655	0,9366	0,1016	-0,64	0,519
	Rapamycin:RU486	0,3189	1,3756	0,1441	2,21	0,027

Genotype/Treatment	Median Lifespan (days)	Maximum Lifespan (days)	n Dead	n Censored	% Increase (med) vs control (Control)	% Increase (max) vs control (Control)	Control	p-value (log rank) 5966GS>Bchs [RNAi] ♂- RU486	5966GS>Bchs [RNAi] ♂- Rapamycin	6GS>Bchs [RNAi] ♂- Rapamycin+RU486
5966GS>Bchs [RNAi] ♂- Control	57	81	194	5			*	0,009501383	0,913960273	0,856038542
5966GS>Bchs [RNAi] ♂- RU486	55	79	195	4	-3,5%	-2,5%			0,005184981	0,020287784
5966GS>Bchs [RNAi] ♂- Rapamycin	57	82	192	6	0,0%	1,2%				0,801323331
966GS>Bchs [RNAi] ♂- Rapamycin+RU48	57	82	193	7	0,0%	1,2%				

Cox Proportional Hazard (CPH) analysis

Details	Coefficient	Coefficient (estimate)	exp(coeff)	SE (coeff)	z	p
dead = 796	Rapamycin	-0,00734	0,99268	0,010188	-0,07	0,09430
censored = 22	RU486	0,26595	1,30467	0,10176	2,61	0,009
	Rapamycin:RU486	-0,24462	0,783	0,14434	-1,69	0,090

Supplementary Table 6 - Knock-down of Bchs in ECs shortened the lifespan only in males (related to Figure 4c,d)

Genotype/Treatment	Median Lifespan	Maximum Lifespan	n Dead	n Censored	% Increase (med)	% Increase (max)	Control	p-value (log rank)		
	(days)	(days)			vs control (Control)	vs control (Control)		5966GS>Bchs [RNAi] ♀- RU486	5966GS>Bchs [RNAi] ♀- Spermidine	6GS>Bchs [RNAi] ♀- Spermidine+RU486
5966GS>Bchs [RNAi] ♀- Control	57	73	188	14			*	0,536001323	0,001298367	0,412805931
5966GS>Bchs [RNAi] ♀- RU486	57	75	196	5	0,0%	2,7%			0,006481928	0,83035697
5966GS>Bchs [RNAi] ♀- Spermidine	60	80	171	28	5,3%	9,6%				0,011558767
966GS>Bchs [RNAi] ♀- Spermidine+RU48	57	78	194	5	0,0%	6,8%				

Cox Proportional Hazard (CPH) analysis

Details	Coefficient	Coefficient (estimate)	exp(coeff)	SE (coeff)	z	p
dead = 801	Spermidine	-0,3543	0,7017	0,1068	-3,32	0,00091
censored = 52	RU486	-0,0534	0,948	0,1022	-0,52	0,602
	Spermidine:RU486	0,3403	1,4053	0,1472	2,31	0,021

Genotype/Treatment	Median Lifespan	Maximum Lifespan	n Dead	n Censored	% Increase (med)	% Increase (max)	Control	p-value (log rank)		
	(days)	(days)			vs control (Control)	vs control (Control)		5966GS>Bchs [RNAi] ♂- RU486	5966GS>Bchs [RNAi] ♂- Spermidine	6GS>Bchs [RNAi] ♂- Spermidine+RU486
5966GS>Bchs [RNAi] ♂- Control	51	73	172	27			*	0,022705589	0,94371045	0,786644509
5966GS>Bchs [RNAi] ♂- RU486	49	78	189	10	-3,9%	6,8%			0,025487925	0,008174014
5966GS>Bchs [RNAi] ♂- Spermidine	51	80	164	35	0,0%	9,6%				0,721598199
966GS>Bchs [RNAi] ♂- Spermidine+RU48	53	75	180	19	3,9%	2,7%				

Cox Proportional Hazard (CPH) analysis

Details	Coefficient	Coefficient (estimate)	exp(coeff)	SE (coeff)	z	p
dead = 796	Spermidine	0,00744	1,00747	0,10927	0,07	0,946
censored = 22	RU486	0,25562	1,29126	0,10605	2,41	0,016
	Spermidine:RU486	-0,28415	0,75265	0,15145	-1,88	0,061

Supplementary Table 7 - Overexpression of TraF in ECs restored the lifespan extension by rapamycin treatment in males (related to Figure 7d)

Genotype/Treatment	Median Lifespan (days)	Maximum Lifespan (days)	n Dead	n Censored	% Increase (med) vs control (Control)	% Increase (max) vs control (Control)	Control	p-value (log rank)		
								mexG4>w - Rapamycin	mexG4>TraF - Control	mexG4>TraF - Rapamycin
mexG4>w - Control	57	82	155	43			*	0,16950250337	0,854170991	2,25808E-05
mexG4>w - Rapamycin	57	79	181	18	0,0%	-3,7%			0,092804711	0,000127203
mexG4>TraF - Control	57	79	141	58			*			1,55333E-06
mexG4>TraF - Rapamycin	63	82	114	85	10,5%	3,8%				

Cox Proportional Hazard (CPH) analysis

Details	Coefficient	Coefficient (estimate)	exp(coeff)	SE (coeff)	z	p
dead = 591	Rapamycin	-0,135	0,8737	0,1104	-1,22	0,2212
censored = 204	TraF	0,0332	1,0337	0,1173	0,28	0,7774
	Rapamycin:TraF	-0,4807	0,6184	0,1716	-2,80	0,0051

Supplementary Table 8 - Knockdown of TraF in ECs blocked the lifespan extension by rapamycin treatment in females (related to Figure 7e)

Genotype/Treatment	Median Lifespan (days)	Maximum Lifespan (days)	n Dead	n Censored	% Increase (med) vs control (Control)	% Increase (max) vs control (Control)	Control	p-value (log rank) mexG4>v - Rapamycin	mexG4>TraF [RNAi] - Control	mexG4>TraF [RNAi] - Rapamycin
mexG4>v - Control	71	86	196	3			*	1,81703E-09	1,56724E-09	7,72704E-12
mexG4>v - Rapamycin	76	91	184	15	7,0%	5,8%			0,915054013	0,466903337
mexG4>TraF [RNAi] - Control	76	89	197	2			*			0,381255525
mexG4>TraF [RNAi] - Rapamycin	76	89	190	9	0,0%	0,0%				

Cox Proportional Hazard (CPH) analysis

Details	Coefficient	Coefficient (estimate)	exp(coeff)	SE (coeff)	z	p
dead = 767	Rapamycin	-0,652	0,521	0,104	-6,25	4.2E-10
censored = 29	TraF [RNAi]	-0,626	0,535	0,102	-6,12	9,50E-10
	Rapamycin:TraF [RNAi]	0,553	1,739	0,145	3,81	0,00014
