# Supplementary material for: Sexual identity of enterocytes regulates autophagy to determine intestinal health, lifespan and responses to rapamycin
Source: Nat Aging. 2022 Dec 1;2(12):1145–58. doi: 10.1038/s43587-022-00308-7 (PMC10154239; doi:10.1038/s43587-022-00308-7)
Supplement: Supplementary file 2 — Reporting Summary [file 43587_2022_308_MOESM2_ESM.pdf]

nature portfolio

Corresponding author(s): Jennifer Regan, Yu-Xuan Lu, Linda Partridge

Last updated by author(s): Oct 3, 2022

Reporting Summary

Nature Portfolio wishes to improve the reproducibility of the work that we publish. This form provides structure for consistency and transparency in reporting. For further information on Nature Portfolio policies, see our Editorial Policies and the Editorial Policy Checklist.

Statistics

For all statistical analyses, confirm that the following items are present in the figure legend, table legend, main text, or Methods section.

n/a	Confirmed	
☐	☒	The exact sample size (*n*) for each experimental group/condition, given as a discrete number and unit of measurement
☐	☒	A statement on whether measurements were taken from distinct samples or whether the same sample was measured repeatedly
☐	☒	The statistical test(s) used AND whether they are one- or two-sided<br>*Only common tests should be described solely by name; describe more complex techniques in the Methods section.*
☐	☒	A description of all covariates tested
☐	☒	A description of any assumptions or corrections, such as tests of normality and adjustment for multiple comparisons
☐	☒	A full description of the statistical parameters including central tendency (e.g. means) or other basic estimates (e.g. regression coefficient) AND variation (e.g. standard deviation) or associated estimates of uncertainty (e.g. confidence intervals)
☐	☒	For null hypothesis testing, the test statistic (e.g. *F*, *t*, *r*) with confidence intervals, effect sizes, degrees of freedom and *P* value noted<br>*Give P values as exact values whenever suitable.*
☒	☐	For Bayesian analysis, information on the choice of priors and Markov chain Monte Carlo settings
☐	☒	For hierarchical and complex designs, identification of the appropriate level for tests and full reporting of outcomes
☒	☐	Estimates of effect sizes (e.g. Cohen's *d*, Pearson's *r*), indicating how they were calculated

Our web collection on statistics for biologists contains articles on many of the points above.

Software and code

Policy information about availability of computer code

Data collection	Leica Application Suite X (LAS X, Leica).<br>ChemiDoc™ XRS+ System + Image Lab v5.1 software, Biorad.<br>QuantStudio™ 6 Flex System + QuantStudio™ Real-time PCR software v.1.7.1, Applied biosystems.<br>Qubit® 3.0 Fluorometer, Fisher Scientific.
Data analysis	IMARIS (v9.1, Oxford Instruments) software was used to quantify lysotracker-stained and Cyto-ID stained puncta.<br>The Fiji (v2.1.0, ImageJ) software package was used to quantify cell size and length of dysplasia.<br>Image Lab (v5.1, Biorad) software was used to quantify protein bands in western blot.<br>GraphPad Prism (v7.0, GraphPad) was used for statistical analysis except for survival data.<br>Excel 2016 (Microsoft) and R-Studio (R v3.5.5) software were used to analyse survival data.<br>Qiime 1 pipeline was used to analyse 16S sequencing data.

For manuscripts utilizing custom algorithms or software that are central to the research but not yet described in published literature, software must be made available to editors and reviewers. We strongly encourage code deposition in a community repository (e.g. GitHub). See the Nature Portfolio guidelines for submitting code & software for further information.

Data

Policy information about availability of data

All manuscripts must include a data availability statement. This statement should provide the following information, where applicable:

- Accession codes, unique identifiers, or web links for publicly available datasets
- A description of any restrictions on data availability
- For clinical datasets or third party data, please ensure that the statement adheres to our policy

The Drosophila melanogaster gut microbiota is publicly available at the NCBI BioProject database (PRJNA877614). All other data of this study are available as Source data files or from the corresponding authors upon reasonable request.

Field-specific reporting

Please select the one below that is the best fit for your research. If you are not sure, read the appropriate sections before making your selection.

☒ Life sciences ☐ Behavioural & social sciences ☐ Ecological, evolutionary & environmental sciences

For a reference copy of the document with all sections, see nature.com/documents/nr-reporting-summary-flat.pdf

Life sciences study design

All studies must disclose on these points even when the disclosure is negative.

Sample size	Sample size for molecular experiments, pathologies analyses and survival analyses were based on prior, published studies by our lab investigating the effects of rapamycin on lifespan, TORC1 inhibition, autophagy induction and gut pathologies (Bjedov et al., 2010; Regan et al.,2016, Lu et al., 2021).
Data exclusions	No data was excluded.
Replication	All survival experiments were performed 2 or 3 times. All attempts at replication were successful, except Fig.4 and Extended Data Fig.2. We did not attempt to replicate the Fig.4. as part of the results just replicated the previous published study (Lu et al. 2021). We did not attempt to replicate the Extended Data Fig. 2 as they served as additional confirmations as Fig. 1a.<br>For each experiment at least 3 biological replicates were used.
Randomization	Upon setting up lifespan and other fly experiments, flies were randomly allocated to different treatments. Upon weaning female mice were randomly assigned to cages.
Blinding	Staining experiments and data analysis were blinded to group allocation during data collection. Other experiments were not possible to blind so they were carried out in an un-blinded fashion unless otherwise stated.

Reporting for specific materials, systems and methods

We require information from authors about some types of materials, experimental systems and methods used in many studies. Here, indicate whether each material, system or method listed is relevant to your study. If you are not sure if a list item applies to your research, read the appropriate section before selecting a response.

Materials & experimental systems

n/a	Involved in the study	
☐	☒	Antibodies
☒	☐	Eukaryotic cell lines
☒	☐	Palaeontology and archaeology
☐	☒	Animals and other organisms
☒	☐	Human research participants
☒	☐	Clinical data
☒	☐	Dual use research of concern

Methods

n/a	Involved in the study	
☒	☐	ChIP-seq
☒	☐	Flow cytometry
☒	☐	MRI-based neuroimaging

Antibodies

Antibodies used	Atg8a (home-made, gift from Péter Nagy's lab, Eötvös Loránd University, Budapest, Hungary (Nagy et al, 2015)<br>Phospho-Drosophila p70 S6 Kinase (Thr398) (Cell Signaling #9209)<br>Total S6K, home-made, from this lab (Bjedov et al, 2010)<br>Histone H3 (Abcam #ab1791)<br>Histone H4 (Active Motif #39269)

Phospho-Histone H3 (Ser10) (Cell Signaling #9701)
p62/SQSTM1 (Abcam, 56416)

Goat Anti-Rabbit IgG Antibody, HRP-conjugate (Sigma, 12-348)
Goat Anti-Mouse IgG Antibody, HRP-conjugate (Sigma, 12-349)
Goat anti-Rabbit IgG (H+L) Cross-Adsorbed Secondary Antibody, Alexa Fluor™ 594 (ThermoFisher Scientific, A-11012)

Validation

Atg8 - validated and published (Nagy et al, 2015)
p-T389-S6K (Cell Signalling Technologies, #9209) - validated by the company and the following publication (Wei et al. 2019)
total S6K - validated and published (Bjedov et al, 2010)
Histone H3 (Abcam #ab1791)- validated by company and the following publication (Zhang et al. 2021)
Histone H4 (Active Motif #39269)- validated by company and the following publication (Ivanov et al. 2013)
p-Histone H3 (Ser10) (Cell Signaling #9701)- validated by the company and the following publication (Dye et al. 2017)
p62/SQSTM1 (Abcam, 56416) - validated by company and the following publication (Koduri et al. 2021)

HRP-conjugate, Goat anti-Rabbit IgG Antibody and Goat Anti-Mouse IgG Antibody (Sigma) - validated by the company and users
Alexa Flour 594 goat anti-rabbit secondary antibody (Thermo Fisher Scientific) - validated by the company and users

Animals and other organisms

Policy information about studies involving animals; ARRIVE guidelines recommended for reporting animal research

Laboratory animals

Drosophila melanogaster: Wolbachia positive males and females were used, unless otherwise stated. Flies were maintained at 25°C on a 12 h light/dark cycle, at constant humidity (60%), and reared on sugar/yeast/agar (SYA) diet. Fly strains used were: Dahomey (Dah) (Broughton et al., 2005), white Dahomey (wDah) (Broughton et al., 2005), UAS-Atg5[RNAi] (Ren et al., 2009), UAS-traF (Bloomington #4590), UAS-traF[RNAi] (Bloomington #44109), mex1Gal4 (Bloomington #91369), 5966GS (Guo et al., 2014), DGRP-OX (Savola et al., 2021), UAS-Bchs[RNAi] (Vienna #KK110785), UAS-H3/H4 (this lab).

Mus musculus: C3B6F1 hybrid mice were generated by a cross between C3H female and C57BL/6J male mice from our in-house animal facility. C3H and C57BL/6J mice were originally from Charles River Laboratories. While females were randomized upon weaning, male mice were weaned litterwise to avoid aggression and fighting. All mice were housed in individually ventilated cages, in groups of five mice per cage, under specific-pathogen-free (SPF) conditions, at 21°C, with 12h light/dark cycle and 50-60% humidity . Mice received a standard rodent diet (Ssniff Spezialdiäten GmbH; 9% fat, 34% protein, 57% carbohydrates) and drinking water at all times. Mice were fasted for 18 hr before euthanasia and tissues were collected, at 12 months of age.

Wild animals

Our study did not involve any wild animals.

Field-collected samples

Our study did not involve any field-collected samples.

Ethics oversight

Mouse experiments were performed in accordance with the recommendations and guidelines of the Federation of the European Laboratory Animal Science Association (FELASA), with all protocols approved by the Landesamt für Natur, Umwelt und Verbraucherschutz, Nordrhein-Westfalen, Germany (reference numbers: 81-02.04.2020.A152).

Note that full information on the approval of the study protocol must also be provided in the manuscript.
